# Integrated Multi-Omics Analysis of the Response of *Lycium barbarum* ‘Ningqi-1’ to Powdery Mildew Infection and Functional Characterization of *LbMLO6*

**DOI:** 10.3390/ijms27219412

**Published:** 2026-10-22

**Authors:** Wendi Xu, Lan Luo, Jiaqi Wang, Ruirui Ren, Jun Zhou, Cuiping Wang, Ken Qin, Yanlong Li, Yunxiang Li, Xin Zhang, Jing Wang, Binbin Si, Linyuan Duan, Guoli Dai

**Affiliations:** 1 School of Biological Science and Engineering, North Minzu University, Yinchuan 750021, China; xuwendi2018@nmu.edu.cn (W.X.);; 2 National Key Laboratory of Crop Genetic Improvement, Huazhong Agricultural University, Wuhan 430070, China; 3 National Wolfberry Engineering Research Center, Ningxia Academy of Agriculture and Forestry Sciences, Yinchuan 750002, China

**Keywords:** *Lycium barbarum*, powdery mildew, multi-omics, *LbMLO6*, CRISPR/Cas9, disease resistance

## Abstract

Powdery mildew severely constrains the production of *Lycium barbarum* L., yet the molecular mechanisms underlying its response to infection remain poorly understood. Here, ‘Ningqi-1’ plants were inoculated with the powdery mildew pathogen and sampled at 0, 12, 24, and 48 hpi for integrated transcriptomic, proteomic, and metabolomic analyses. Quantitative PCR and CRISPR/Cas9-mediated gene editing were further used to evaluate the candidate susceptibility gene *LbMLO6*. Infection induced extensive molecular reprogramming, yielding 1299 differentially expressed genes with sustained changes, 75 DAPs shared across all three time points, and 127 shared differentially accumulated metabolites. These molecules were mainly enriched in plant–pathogen interaction, plant hormone signal transduction, MAPK signaling, phenylpropanoid biosynthesis, and α-linolenic acid metabolism pathways, suggesting coordinated roles for Ca^2+^ signaling, reactive oxygen species, defense-related proteins, and secondary metabolism in the host response. Integrated multi-omics analysis identified *LbMLO6* as a candidate gene associated with powdery mildew susceptibility, its expression was markedly induced from 24 hpi onward. CRISPR/Cas9-edited *LbMLO6* lines showed reduced fungal development compared with the control, particularly during the early stages of infection. These findings provide preliminary evidence that *LbMLO6* may be associated with powdery mildew susceptibility in *L. barbarum*.

## 1. Introduction

Goji berry (*Lycium barbarum* L.), a perennial deciduous shrub belonging to the Solanaceae family, is widely cultivated in arid and semi-arid regions because of its strong adaptability to drought, salinity, and nutrient-poor soils [1,2]. Its fruits and vegetative tissues contain diverse bioactive compounds and have considerable nutritional and medicinal value. However, goji berry production is continuously challenged by various biotic and abiotic stresses. In particular, changes in cultivation practices, climate, and agroecological conditions have contributed to increasingly frequent and severe disease outbreaks, posing a major threat to sustainable goji berry production. Among these diseases, powdery mildew (PM) is one of the most destructive fungal diseases affecting goji berry.

Powdery mildew is caused by obligate biotrophic fungi that obtain nutrients from living host cells through specialized feeding structures known as haustoria. Collectively, powdery mildew fungi infect more than 10,000 plant species worldwide [3,4]. More than 10 diseases have been reported in goji berry, among which powdery mildew is considered one of the most damaging fungal diseases [5]. In goji berry, *Arthrocladiella mougeotii* has been reported as a causal agent of powdery mildew, which can severely affect leaf function and plant growth and ultimately reduce yield and fruit quality. Understanding the molecular responses of goji berry to powdery mildew infection and identifying key genes associated with disease resistance are therefore important for elucidating goji berry–powdery mildew interactions and developing resistant germplasm.

Among the genes associated with powdery mildew resistance, mildew resistance locus O (*MLO*) genes have received considerable attention because loss-of-function mutations in specific *MLO* members can confer broad-spectrum and durable resistance. *MLO* was first identified as a powdery mildew susceptibility gene in barley (*Hordeum vulgare* L.). Unlike resistance mediated by conventional dominant resistance (*R*) genes, recessive loss-of-function *mlo* alleles can confer effective and durable resistance to powdery mildew [6,7,8]. MLO proteins are plant-specific seven-transmembrane proteins with an extracellular N-terminus and an intracellular C-terminus containing a calmodulin-binding domain. Recent studies have further indicated that some MLO proteins possess Ca^2+^ channel activity, suggesting a potential link between MLO proteins and calcium-dependent signaling involved in plant immunity [9,10].

Genome-wide analyses have revealed that *MLO* genes generally occur as multigene families in higher plants, although their gene numbers, phylogenetic relationships, and functional differentiation vary among species. The *MLO* gene family has been systematically characterized in numerous plant species, including members of the Cucurbitaceae, Rosaceae, and Poaceae [11,12,13,14]. Genetic analysis, gene silencing, and genome editing have demonstrated that specific *MLO* members are associated with powdery mildew susceptibility in both monocotyledonous and dicotyledonous plants, including *Arabidopsis*, grapevine, rose, and wheat [15,16,17,18]. For example, Kim et al. identified a novel *Slmlo1.1* allele in a powdery mildew-resistant tomato line, in which a 1 bp deletion resulted in a frameshift mutation associated with loss of *SlMlo1* function and enhanced resistance [19]. Downregulation of *MdMLO19* reduced powdery mildew susceptibility in apple [20], while simultaneous editing of *VvMLO6* and *VvMLO7* enhanced resistance in grapevine [21]. Similarly, 14 *MLO* family members were identified in melon, and *CmMLO5* was proposed as a susceptibility-associated gene involved in the melon–powdery mildew interaction [22]. Collectively, these studies demonstrate that specific *MLO* genes play conserved roles in powdery mildew susceptibility across diverse plant species. However, the *MLO* gene family in goji berry and the potential involvement of its members in the response to powdery mildew infection remain poorly understood.

Advances in high-throughput sequencing and mass spectrometry have enabled integrated multi-omics approaches to provide a more comprehensive view of plant–pathogen interactions. Whereas individual omics approaches capture molecular changes at a specific regulatory level, integration of transcriptomic, proteomic, and metabolomic datasets enables coordinated characterization of changes in gene expression, protein abundance, and metabolite accumulation [23]. Cross-omics correlation analysis can further facilitate the identification of candidate regulatory genes, proteins, metabolites, and biological pathways associated with disease responses. For example, integrated transcriptomic and metabolomic analysis of strawberry infected with powdery mildew revealed substantial changes in plant–pathogen interaction, carbon metabolism, and photosynthesis-related pathways and identified potentially important metabolites, including quercetin [24]. Similarly, integrated transcriptomic, proteomic, and metabolomic analyses of powdery mildew-resistant and susceptible barley varieties revealed coordinated molecular changes associated with resistance and highlighted jasmonic acid-related pathways and phenolamides as potentially important components of the response [25]. Metabolomic and microbiome analyses of pathogen-infected wheat have also revealed tissue-specific associations between plant metabolites and microbial communities [26]. These findings demonstrate the value of multi-omics integration for identifying molecular components and pathways potentially associated with plant responses to pathogen infection.

Genome-editing technologies provide powerful tools for the functional validation of candidate genes and the development of disease-resistant germplasm. Among currently available genome-editing systems, including zinc finger nucleases (ZFNs), transcription activator-like effector nucleases (TALENs), and CRISPR/Cas systems [27,28], CRISPR/Cas9 has been widely adopted because of its relatively simple design, high efficiency, and capacity for targeted genome modification [29]. It has been successfully applied to the improvement of important agronomic traits, including yield, quality, and disease resistance. For example, targeted modification of *TaGW2* alters grain size and yield-related traits in wheat [30,31], whereas CRISPR/Cas9-mediated knockout of *SlMlo1* confers enhanced resistance to powdery mildew in tomato [32]. Furthermore, targeted editing of *CaMLO2* in six commercial pepper cultivars demonstrated the feasibility of modifying *MLO* susceptibility genes across different genetic backgrounds [33]. These findings highlight *MLO* genes as promising targets for functional validation and molecular breeding for powdery mildew resistance. However, whether specific *MLO* members contribute to powdery mildew susceptibility in goji berry and whether targeted modification of these genes can alter its response to powdery mildew infection remain to be determined.

In this study, the goji berry cultivar Ningqi-1 was used to investigate the molecular response to powdery mildew infection and to identify key genes associated with disease resistance. Transcriptomic, proteomic, and metabolomic datasets were integrated to characterize differentially expressed genes (DEGs), differentially abundant proteins (DAPs), and differentially accumulated metabolites (DAMs), and to explore their cross-omics associations. Based on the multi-omics analysis, candidate *MLO* genes potentially involved in the response to powdery mildew were further identified and systematically characterized in terms of their physicochemical properties, structural features, phylogenetic relationships, and expression patterns. Subsequently, CRISPR/Cas9-mediated genome editing was employed to generate mutant lines of the selected candidate gene, and the resulting mutants were challenged with powdery mildew to evaluate their disease responses and further investigate the role of the candidate gene in powdery mildew resistance. By integrating multi-omics analysis, candidate gene characterization, genome editing, and disease-response evaluation, this study provides new insights into the molecular basis of the goji berry–powdery mildew interaction and provides a theoretical and genetic foundation for the development of powdery mildew-resistant goji berry germplasm.

## 2. Results

### 2.1. Transcriptome Analysis of Ningqi-1 in Response to Infection by the Powdery Mildew Fungus

#### 2.1.1. Transcriptome Analysis, PCA (Principal Component) Analysis, and Sample Correlation Analysis

Transcriptome sequencing was performed on *Lycium barbarum* leaves from four groups, including the CK control and samples collected at 12, 24, and 48 h post-inoculation (hpi), with three biological replicates per group, resulting in a total of 12 samples. After quality filtering, clean reads were obtained from all samples. The GC content ranged from 42.25% to 43.43%, with Q20 values above 97% and Q30 values above 92%, and the highest Q30 value reached 93.02% (Appendix A Table A8). The clean reads were subsequently aligned to the *L. barbarum* reference genome. The mapping rates of all samples exceeded 70%, with CK-2 showing the highest mapping rate of 81.51% (Appendix A Table A9). Notably, the 48 h samples showed lower mapping rates than the CK samples, although Q30 values remained above 92% for all samples. Reads were aligned only to the *L. barbarum* reference genome, and unmapped reads were not further classified. The source of the lower mapping rates at 48 h therefore remains unresolved.

**Figure 1:**
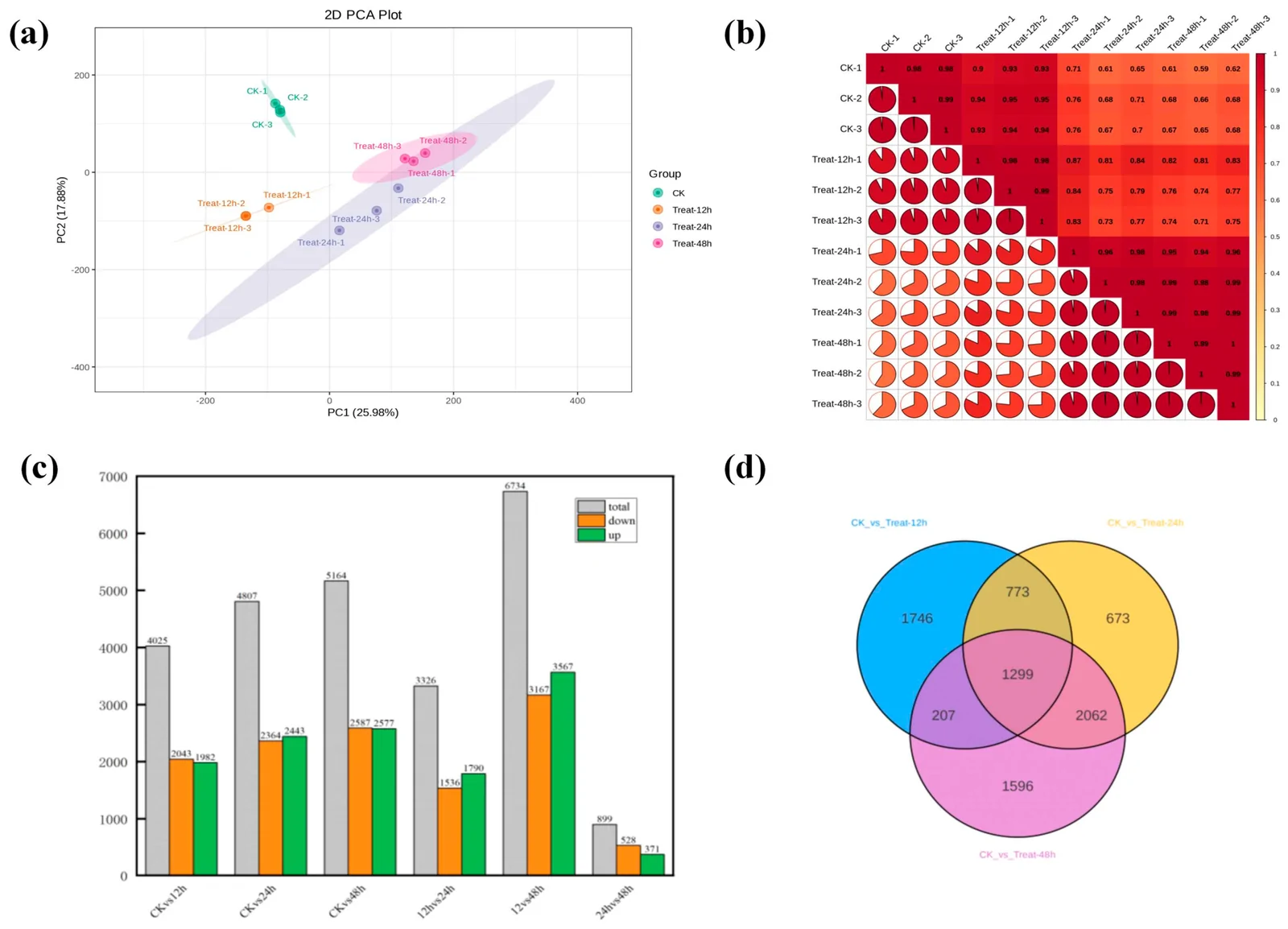
Transcriptomic characteristics and differential gene expression in *Lycium barbarum* following powdery mildew inoculation. (**a**) Principal component analysis (PCA) of the 12 RNA-seq samples. (**b**) Pearson correlation analysis among the 12 RNA-seq samples. (**c**) Numbers of total, up-regulated, and down-regulated differentially expressed genes (DEGs) in pairwise comparisons among CK, 12, 24, and 48 h post-inoculation (hpi). (**d**) Venn diagram showing shared and comparison-specific DEGs among the CK vs. 12 h, CK vs. 24 h, and CK vs. 48 h comparisons.

Principal component analysis (PCA) was performed to summarize the major sources of variation in global gene expression across the 12 RNA-seq samples (Figure 1a). PC1 and PC2 accounted for 25.98% and 17.88% of the total variance, respectively, together explaining 43.86% of the overall variation. whereas PC3 and PC4 contributed an additional 8.89% and approximately 7%, respectively, bringing the cumulative variance of the first four PCs to ~60% (Figure S1). The four sampling groups showed different distribution patterns in the PCA space. Biological replicates from the CK, 12 h, and 48 h groups clustered relatively closely, whereas the 24 h replicates were more dispersed, suggesting greater within-group variation at this time point. The 24 h and 48 h samples were located relatively close to one another along PC1 and PC2, indicating similarity in the major sources of variation represented by these two principal components. Pearson correlation analysis was further performed to evaluate sample consistency (Figure 1b). Relatively high correlations were observed among biological replicates within the same group, supporting the overall reproducibility of the RNA-seq dataset. Collectively, these results indicated satisfactory overall sample reproducibility, although greater biological variation was observed among the 24 h replicates.

#### 2.1.2. Analysis of Differentially Expressed Genes

To investigate the transcriptional responses of *Lycium barbarum* leaves to powdery mildew infection at different time points, differentially expressed genes (DEGs) were identified through pairwise comparisons among the CK, 12 h, 24 h, and 48 h groups (Figure 1c). Compared with CK, a total of 4025 DEGs were identified at 12 h, including 1982 up-regulated and 2043 down-regulated genes. At 24 h, 4807 DEGs were detected, including 2443 up-regulated and 2364 down-regulated genes. At 48 h, 5164 DEGs were identified, including 2577 up-regulated and 2587 down-regulated genes. Thus, the number of DEGs relative to CK increased from 12 to 48 h after inoculation. Pairwise comparisons among the three post-inoculation time points further revealed temporal changes in gene expression. A total of 3326 DEGs were identified between 12 h and 24 h, including 1790 up-regulated and 1536 down-regulated genes. The largest number of DEGs was observed between 12 h and 48 h, with 6734 DEGs, including 3567 up-regulated and 3167 down-regulated genes. In contrast, 899 DEGs were detected between 24 h and 48 h, including 371 up-regulated and 528 down-regulated genes. These results indicate dynamic changes in the transcriptomic response of *L. barbarum* leaves during powdery mildew infection. Venn analysis of the CK vs. 12 h, CK vs. 24 h, and CK vs. 48 h comparisons identified 1299 DEGs shared among all three comparisons (Figure 1d). In addition, 1746, 673, and 1596 DEGs were specific to the CK vs. 12 h, CK vs. 24 h, and CK vs. 48 h comparisons, respectively. These results indicate that powdery mildew infection induced both shared and time-specific transcriptional responses in *L. barbarum* leaves.

#### 2.1.3. GO Functional Classification and KEGG Pathway Enrichment Analysis

**Figure 2:**
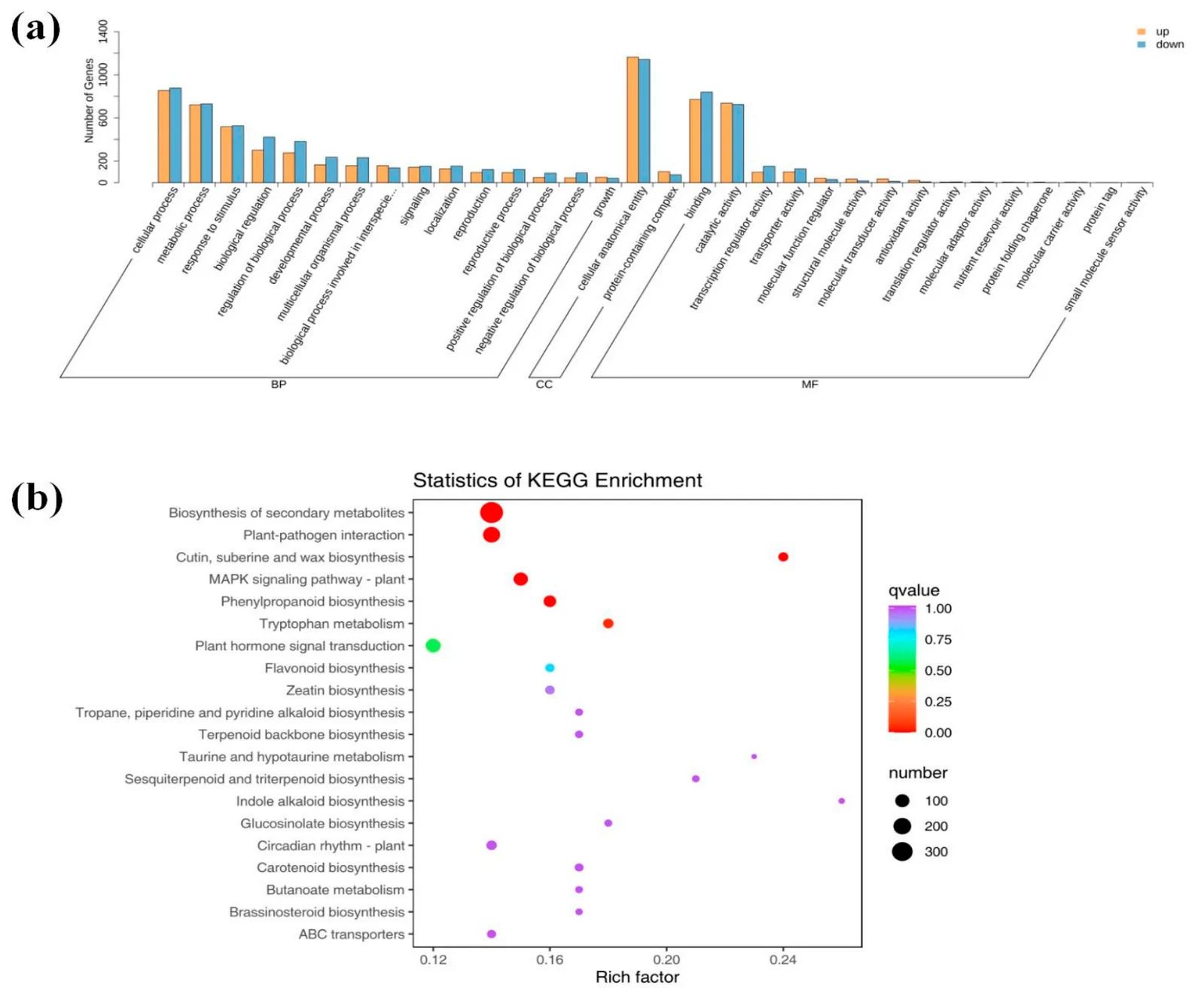
GO functional classification and KEGG pathway enrichment analysis of differentially expressed genes (DEGs) between CK and 12 h post-inoculation (hpi). (**a**) GO functional classification of DEGs into Biological Process (BP), Cellular Component (CC), and Molecular Function (MF) categories. (**b**) KEGG pathway enrichment analysis of DEGs. The bubble size represents the number of DEGs assigned to each pathway, the color represents the *q*-value, and the Rich factor indicates the degree of pathway enrichment.

Gene Ontology (GO) functional classification analysis was performed for the differentially expressed genes (DEGs) identified at different time points following powdery mildew inoculation. The DEGs were classified into three major GO categories: Biological Process (BP), Cellular Component (CC), and Molecular Function (MF) (Figure 2a and Supplementary Figure S1). Among the top 30 GO terms, 15, 2, and 13 terms belonged to the BP, CC, and MF categories, respectively. Across the different time point comparisons, “cellular process”, “metabolic process”, and “response to stimulus” were the major terms in the BP category. In the CC category, DEGs were predominantly assigned to “cellular anatomical entity” and “protein-containing complex”, whereas “binding”, “catalytic activity”, and “transcription regulator activity” were the major terms in the MF category. Although the numbers of up- and down-regulated genes assigned to individual GO terms varied among comparisons, the overall functional classification patterns were broadly similar, indicating that the transcriptional response to powdery mildew involved multiple biological processes, cellular components, and molecular functions.

KEGG pathway enrichment analysis was further performed to identify pathways associated with the response of *Lycium barbarum* to powdery mildew. After multiple-testing correction, pathways with an FDR < 0.05 were considered significantly enriched, and a total of 39 significantly enriched pathways were identified (Appendix A Table A10). The top 20 pathways are presented in Figure 2b and Supplementary Figure S2. Among these, several pathways associated with plant defense responses were identified, including “plant–pathogen interaction”, “MAPK signaling pathway–plant”, “plant hormone signal transduction”, “phenylpropanoid biosynthesis”, “flavonoid biosynthesis,” and “cutin, suberine and wax biosynthesis”. In addition, “biosynthesis of secondary metabolites” and several other metabolic and biosynthetic pathways were represented among the top pathways. These results indicate that the response of *L. barbarum* to powdery mildew involves coordinated changes in defense signaling and secondary metabolic processes. Based on their relevance to plant defense responses, the plant hormone signal transduction and plant–pathogen interaction pathways were selected for further analysis.

#### 2.1.4. Expression Patterns of Differentially Expressed Genes Involved in Plant–Pathogen Interaction and Plant Hormone Signaling Pathways

**Figure 3:**
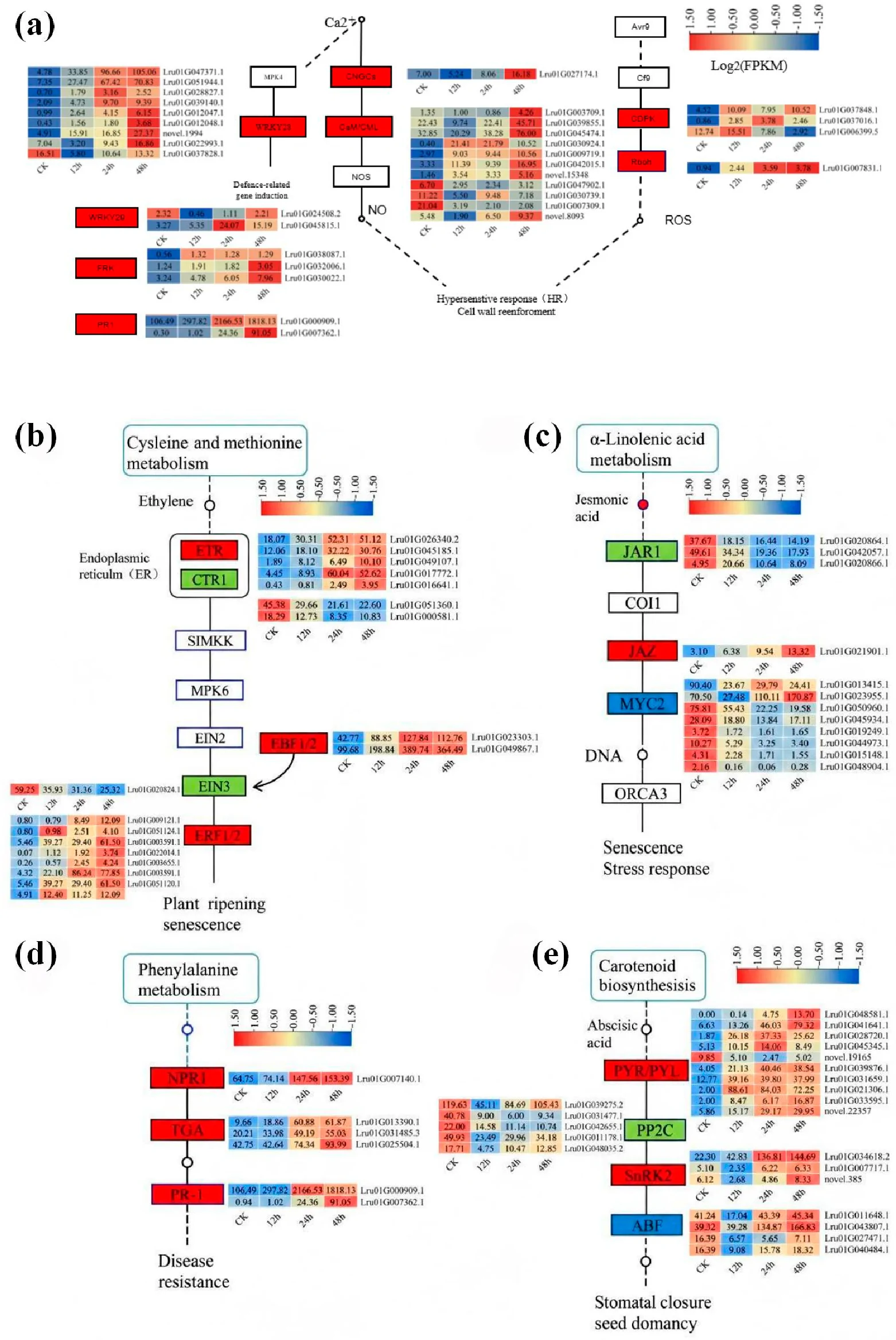
Expression patterns of differentially expressed genes involved in plant–pathogen interaction and plant hormone signaling pathways. (**a**) Plant–pathogen interaction pathway; (**b**) ethylene (ET) signaling pathway; (**c**) jasmonic acid (JA) signaling pathway; (**d**) salicylic acid (SA) signaling pathway; (**e**) abscisic acid (ABA) signaling pathway. Heatmaps show the expression levels (FPKM) of the corresponding genes at CK, 12, 24, and 48 h post-inoculation (hpi), with colors representing log_2_ (FPKM) values.

Based on the KEGG enrichment results, differentially expressed genes (DEGs) involved in the plant–pathogen interaction pathway were further examined (Supplementary Figure S3), with particular attention to genes potentially involved in the response to powdery mildew (Figure 3a). Several genes associated with pathogen recognition and downstream defense signaling showed marked changes in expression following inoculation. One CNGC gene showed decreased expression at 12 h, followed by increased expression at later time points, reaching approximately 2.3-fold the CK level at 48 h. Genes encoding CaM/CML proteins also exhibited dynamic expression patterns, with several genes reaching their highest expression levels at 12 or 24 h. Defense-related transcription factors, including *WRKY28* and *WRKY29*, also showed substantial changes in expression during the response. *FRK* genes exhibited increased expression at later stages of inoculation, while two *PR-1* genes showed particularly strong induction, reaching approximately 17- and 303-fold the CK levels, respectively. In addition, genes encoding CDPKs and Rboh showed altered expression patterns. Collectively, these expression changes suggest the involvement of Ca^2+^ signaling, WRKY-mediated transcriptional regulation, and ROS-associated signaling in the defense response of *Lycium barbarum* to powdery mildew.

To further investigate hormone-related responses to powdery mildew, DEGs involved in ethylene (ET), jasmonic acid (JA), salicylic acid (SA), and abscisic acid (ABA) signaling pathways were analyzed (Figure 3b–e and Supplementary Figure S4). In the ET signaling pathway (Figure 3b), genes encoding ethylene receptors (ETRs), EBF1/2, and ERF1/2 exhibited dynamic expression patterns following inoculation, while several CTR1- and EIN3-related genes also showed marked changes in expression. In the JA signaling pathway (Figure 3c), *JAR1* genes involved in JA-Ile formation showed altered expression, while *JAZ* expression increased during the later stages of inoculation. In contrast, most *MYC2*-related genes showed decreased expression relative to CK, although their expression patterns varied among individual genes. In the SA signaling pathway (Figure 3d), genes encoding NPR1, TGA transcription factors, and *PR-1* exhibited pronounced transcriptional responses, particularly at 24 and 48 h. Notably, the *PR-1* genes showed strong induction at 48 h, as described above. In the ABA signaling pathway (Figure 3e), genes encoding PYR/PYL receptors, PP2Cs, SnRK2s, and ABFs displayed distinct temporal expression patterns, with substantial variation among individual gene family members. Taken together, these results indicate that multiple hormone signaling pathways, particularly ET, JA, SA, and ABA signaling, are transcriptionally modulated during the response of *L. barbarum* to powdery mildew.

#### 2.1.5. RT-qPCR Validation of RNA-Seq Data

**Figure 4:**
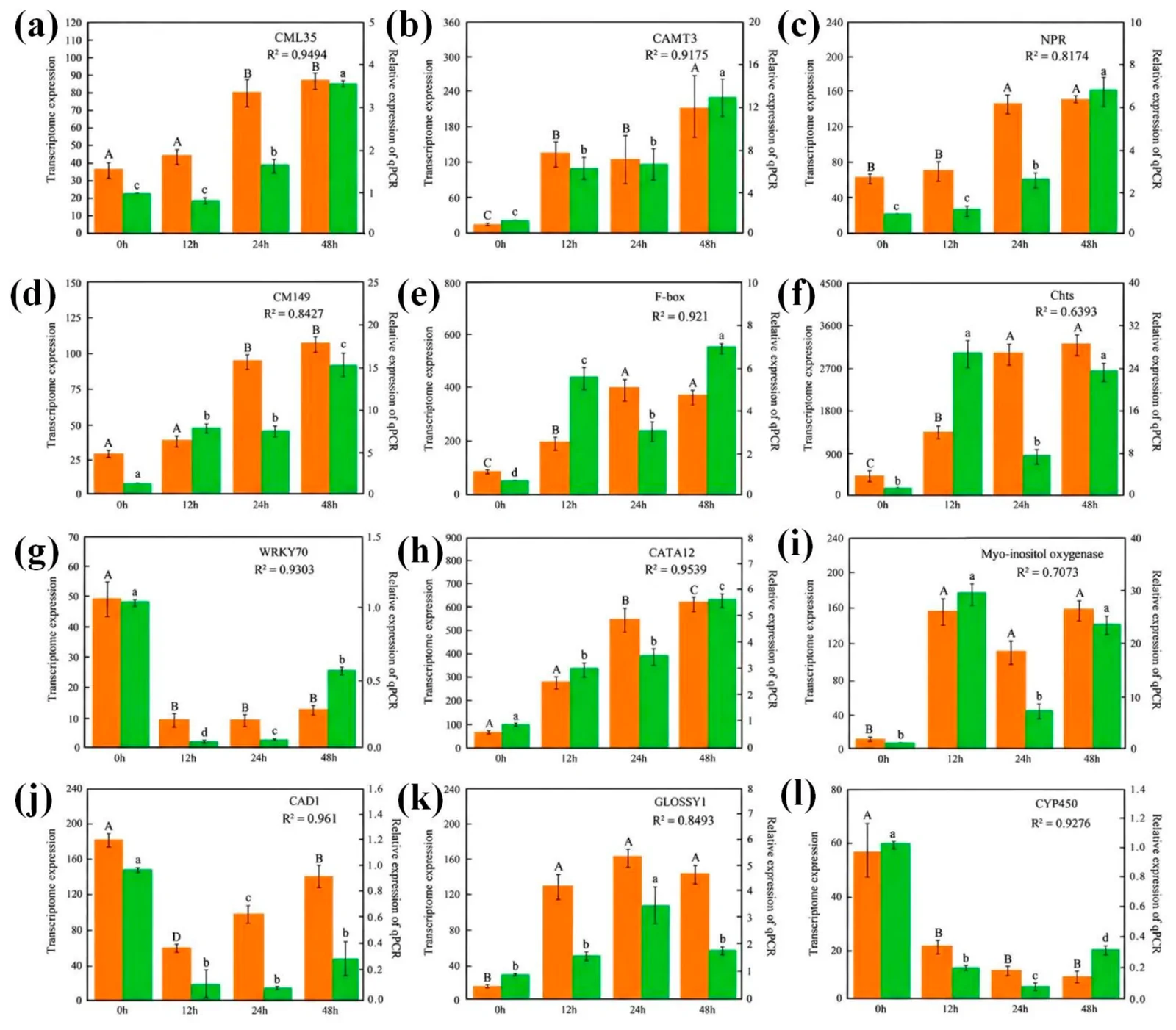
RT-qPCR validation of RNA-seq expression patterns. (**a**–**l**) Uppercase letters indicate RNA-seq data; lowercase letters indicate qPCR data. Statistical significance was determined by one-way ANOVA with Tukey’s test. Expression patterns of 12 selected DEGs determined by RNA-seq and RT-qPCR at 0, 12, 24, and 48 h post-inoculation (hpi). Orange bars represent RNA-seq expression levels, and green bars represent relative expression levels determined by RT-qPCR. Error bars indicate mean ± SD. Different uppercase and lowercase letters indicate significant differences among time points for RNA-seq and RT-qPCR data, respectively (*p* < 0.05). R^2^ represents the coefficient of determination between the RNA-seq and RT-qPCR expression patterns.

To validate the gene expression patterns obtained from RNA-seq analysis, 12 representative DEGs associated with plant defense responses were selected for RT-qPCR analysis, including CML35, CMl49, WRKY70, NPR, Chts, F-box, CATA12, CYP450, GLOSSY1, MIOX (Myo-inositol oxygenase), CAD1, and CAMT3 (Figure 4a–l). Overall, the expression patterns obtained by RT-qPCR were largely consistent with those observed in the RNA-seq data. The coefficients of determination (R^2^) between the RNA-seq and RT-qPCR expression patterns ranged from 0.6393 to 0.9610. Except for Chts (R^2^ = 0.6393), all validated genes showed R^2^ values greater than 0.7, with CAD1 showing the highest value (R^2^ = 0.9610). These results demonstrate a high degree of consistency between the RT-qPCR and RNA-seq expression patterns, supporting the reliability of the RNA-seq data.

### 2.2. Proteomic Data Analysis of Ningqi-1 Response to Powdery Mildew Infection

#### 2.2.1. Proteomic Data Quality Assessment and Sample Reproducibility Analysis

**Figure 5:**
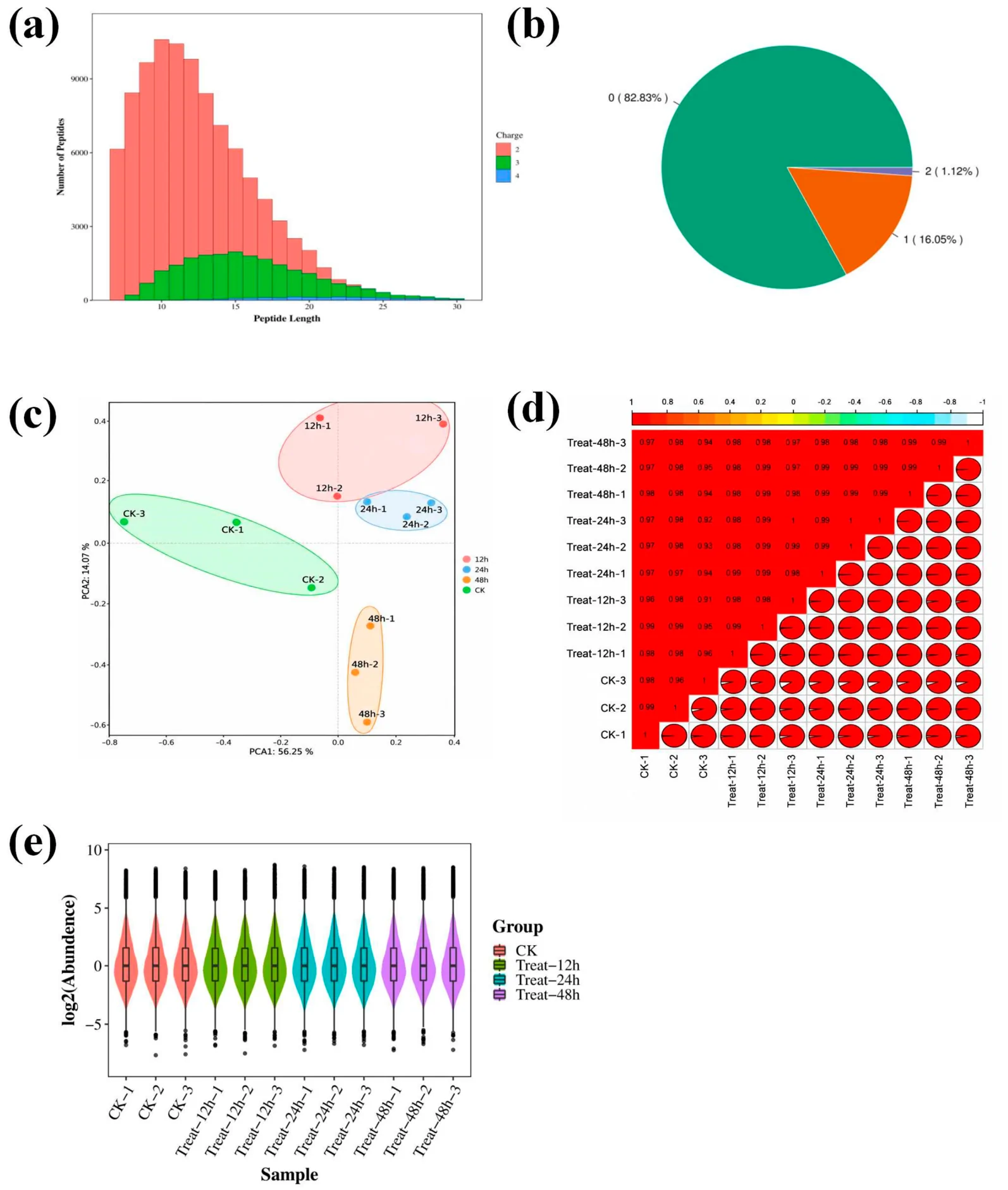
Quality assessment and sample reproducibility analysis of the proteomic data. (**a**) Peptide length distribution. (**b**) Distribution of missed cleavage sites in identified peptides. (**c**) Principal component analysis (PCA) of the 12 proteomic samples. (**d**) Pearson correlation analysis among the 12 samples. (**e**) Distribution of protein abundance [log_2_(Abundance)] across samples.

The quality of the proteomic data was first evaluated based on peptide length distribution and missed cleavage sites. As shown in Figure 5a, the identified peptides were predominantly distributed between 7 and 20 amino acids in length, indicating an appropriate peptide length distribution for subsequent proteomic analysis. Analysis of missed cleavage sites showed that 82.83% of the identified peptides had no missed cleavage sites, whereas 16.05% and 1.12% contained one and two missed cleavage sites, respectively (Figure 5b). These results indicated efficient enzymatic digestion and satisfactory overall quality of the proteomic data.

Sample reproducibility was further evaluated among the 12 samples from the four groups. Principal component analysis (PCA) showed distinct clustering patterns among the four groups (Figure 5c). The CK samples were clearly separated from the three post-inoculation groups, while biological replicates within each group generally clustered together. PC1 and PC2 explained 56.25% and 14.07% of the total variation, respectively, accounting for 70.32% of the total variation. Pearson correlation analysis further showed high correlations among biological replicates within each group, with correlation coefficients generally exceeding 0.90 (Figure 5d). In addition, the distributions of protein abundance were broadly similar among biological replicates within each group (Figure 5e). Collectively, these results indicated good reproducibility among biological replicates and supported the suitability of the proteomic data for subsequent differential protein expression analysis.

#### 2.2.2. Identification of Differentially Expressed Proteins

**Figure 6:**
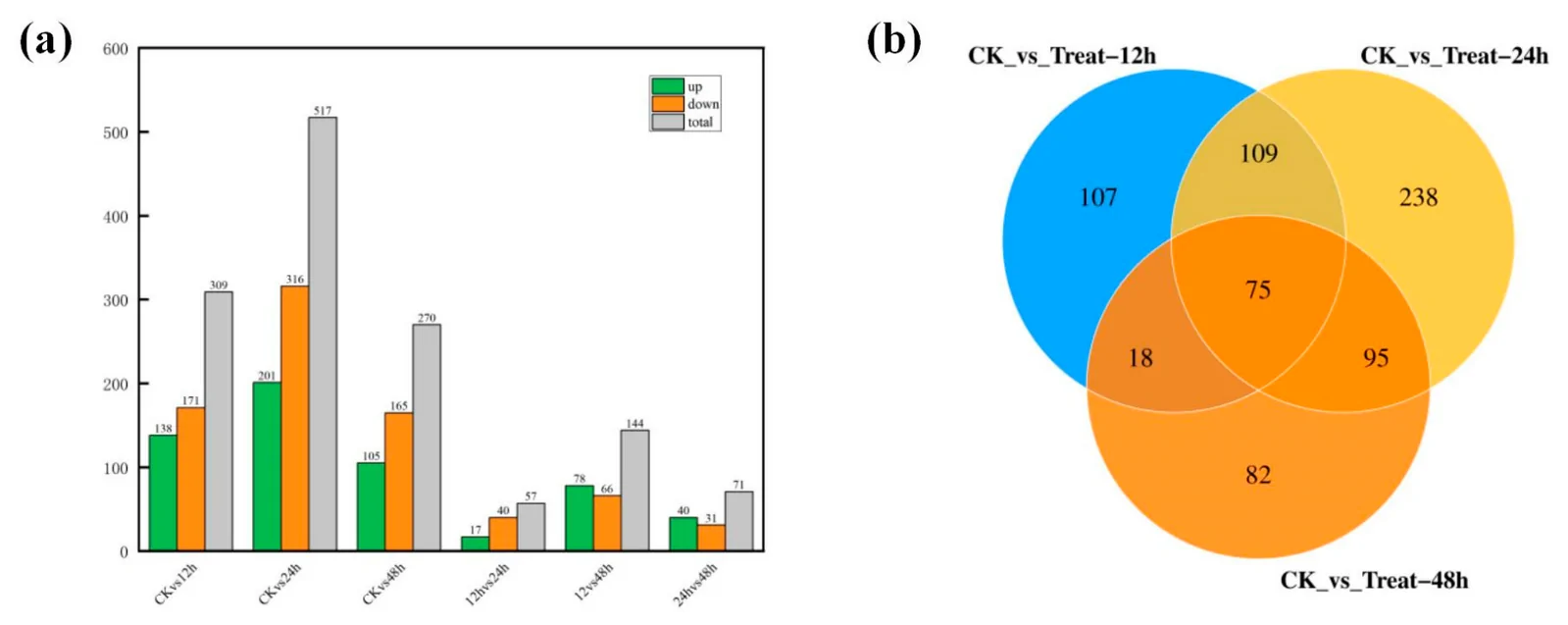
Differentially expressed proteins (DEPs) in *Lycium barbarum* following powdery mildew inoculation. (**a**) Numbers of total, up-regulated, and down-regulated DEPs in pairwise comparisons among CK, 12, 24, and 48 h post-inoculation (hpi). (**b**) Venn diagram showing shared and comparison-specific DEPs among the CK vs. 12 h, CK vs. 24 h, and CK vs. 48 h comparisons.

Differentially expressed proteins (DEPs) were defined as proteins with Benjamini–Hochberg adjusted *p*-value (FDR) < 0.05 and fold change (FC) > 1.5 or FC < 0.67. As shown in Figure 6a, 309 DEPs were identified between CK and 12 h, including 138 up-regulated and 171 down-regulated proteins. Between CK and 24 h, 517 DEPs were identified, including 201 up-regulated and 316 down-regulated proteins. Between CK and 48 h, 270 DEPs were identified, comprising 105 up-regulated and 165 down-regulated proteins. Pairwise comparisons among the three post-inoculation time points identified 57 DEPs between 12 and 24 h, including 17 up-regulated and 40 down-regulated proteins; 144 DEPs between 12 and 48 h, including 78 up-regulated and 66 down-regulated proteins; and 71 DEPs between 24 and 48 h, including 40 up-regulated and 31 down-regulated proteins.

Venn analysis of the CK vs. 12 h, CK vs. 24 h, and CK vs. 48 h comparisons identified 75 DEPs shared among all three comparisons (Figure 6b). In addition, 107, 238, and 82 DEPs were specific to the CK vs. 12 h, CK vs. 24 h, and CK vs. 48 h comparisons, respectively. These results indicate both shared and time-specific changes in protein abundance in response to powdery mildew inoculation.

#### 2.2.3. GO and KOG Functional Classification and KEGG Pathway Enrichment Analysis

**Figure 7:**
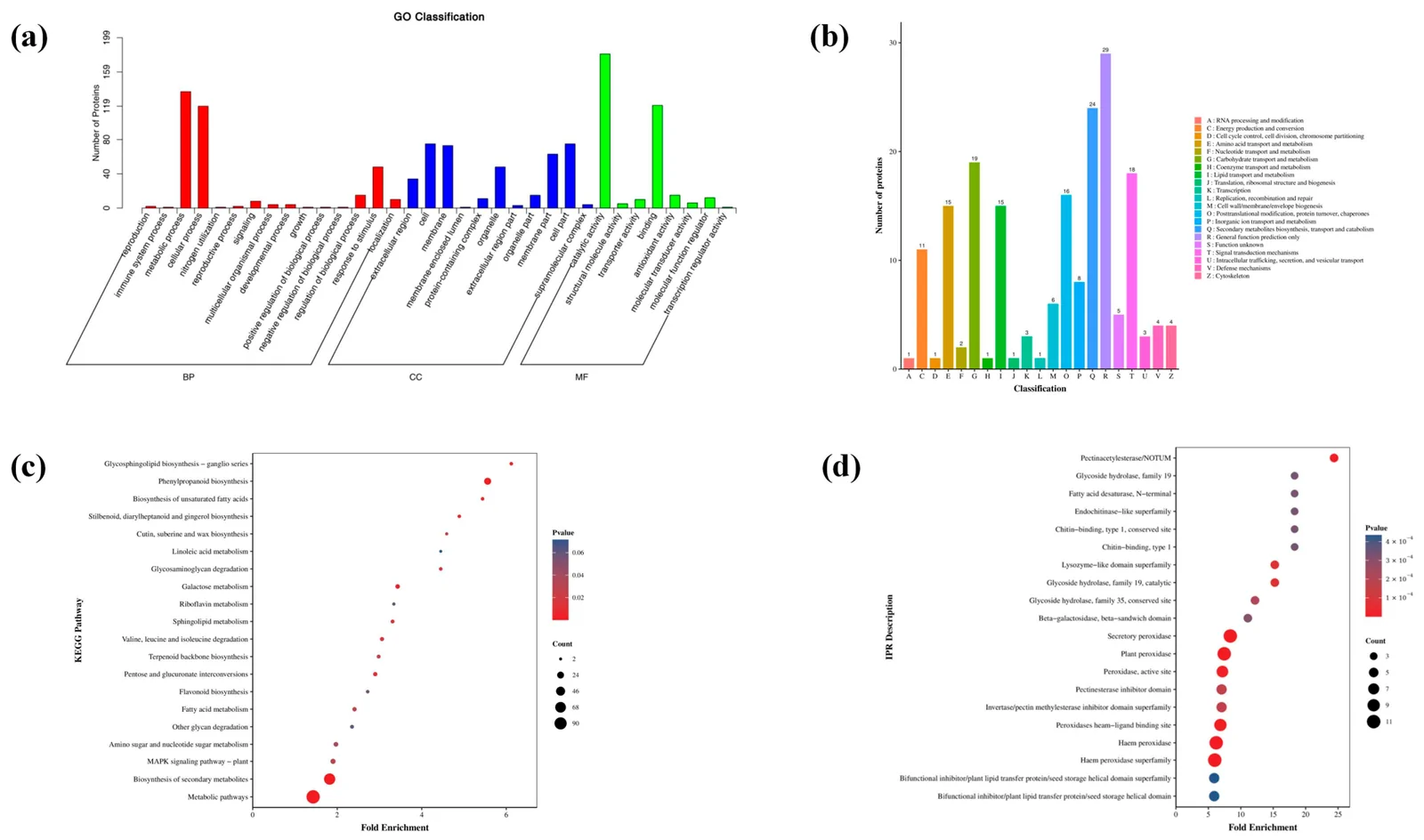
Functional classification and enrichment analysis of differentially expressed proteins (DEPs) between CK and 12 h post-inoculation (hpi). (**a**) Gene Ontology (GO) functional classification of DEPs into Biological Process (BP), Cellular Component (CC), and Molecular Function (MF) categories. (**b**) KOG functional classification of DEPs. (**c**) KEGG pathway enrichment analysis of DEPs; the top 20 pathways ranked by *p*-value are shown. Bubble size represents the number of DEPs, and color represents the *p*-value. (**d**) InterPro (IPR) domain enrichment analysis of DEPs; the top 20 entries ranked by *p*-value are shown. Bubble size represents the number of DEPs, and color represents the *p*-value.

Gene Ontology (GO) functional classification was performed for the differentially expressed proteins (DEPs) identified at different time points following powdery mildew inoculation (Figure 7a and Supplementary Figure S5). The DEPs were classified into three major GO categories: Biological Process (BP), Cellular Component (CC), and Molecular Function (MF). In the BP category, “metabolic process”, “cellular process,” and “response to stimulus” contained relatively large numbers of DEPs. In the CC category, DEPs were mainly assigned to “cell”, “membrane”, “membrane-enclosed lumen”, “protein-containing complex”, and “organelle”. In the MF category, “catalytic activity” and “binding” contained the largest numbers of DEPs. Overall, the GO functional classification patterns were broadly similar among the different time point comparisons, although the numbers of DEPs assigned to individual GO terms varied.

KOG functional classification was further performed to characterize the functions of the DEPs (Figure 7b and Supplementary Figure S6). In the CK vs. 12 h comparison, the major KOG categories included “general function prediction only”, “posttranslational modification, protein turnover, chaperones”, “carbohydrate transport and metabolism”, “amino acid transport and metabolism”, and “secondary metabolites biosynthesis, transport and catabolism”. The numbers of DEPs assigned to these categories were 29, 24, 19, 15, and 18, respectively. The KOG classification profiles varied among the different time point comparisons, reflecting temporal differences in the functional distribution of DEPs following powdery mildew inoculation.

KEGG pathway enrichment analysis was subsequently performed to identify pathways associated with the proteomic response to powdery mildew (Figure 7c and Supplementary Figure S7). Pathways with an FDR < 0.05 were considered significantly enriched, and the top 20 significantly enriched pathways ranked by *p*-value were visualized. In the CK vs. 12 h comparison, DEPs were enriched in several metabolic and defense-related pathways, including “metabolic pathways”, “biosynthesis of secondary metabolites”, “MAPK signaling pathway–plant”, “phenylpropanoid biosynthesis”, “flavonoid biosynthesis”, “cutin, suberine, and wax biosynthesis,” and “linoleic acid metabolism”. The enrichment profiles differed among the three post-inoculation time points, indicating temporal changes in protein abundance associated with metabolic and defense-related processes during the response of *Lycium barbarum* to powdery mildew.

#### 2.2.4. Protein Domain Enrichment Analysis

Protein domain enrichment analysis of the DEPs was performed using InterPro (IPR) annotations (Figure 7d and Supplementary Figure S8). The top 20 enriched IPR entries ranked by *p*-value were visualized as bubble plots. In the CK vs. 12 h comparison, enriched domains included “pectinacetylesterase/NOTUM”, “glycoside hydrolase family 19”, “fatty acid desaturase, N-terminal”, “endochitinase-like superfamily”, “chitin-binding type I conserved site”, “chitin-binding type I”, “lysozyme-like domain superfamily”, “glycoside hydrolase family 19, catalytic”, “glycoside hydrolase family 35, conserved site”, “β-galactosidase β-sandwich domain,” and several peroxidase-related domains. In addition, pectinesterase inhibitor-related and bifunctional inhibitor/plant lipid transfer protein/seed storage helical domain superfamily entries were enriched. These results indicate that proteins associated with carbohydrate metabolism, cell-wall-related processes, oxidative responses, and defense-related functions were represented among the enriched protein domains following powdery mildew inoculation. Peroxidase enzymatic activity was not measured in this study; the proteomic results therefore describe changes in protein abundance and domain enrichment rather than catalytic activity.

### 2.3. Metabolomic Analysis of ‘Ningqi-1’ in Response to Powdery Mildew Infection

#### 2.3.1. Metabolomic Profiling and Sample Reproducibility Analysis

**Figure 8:**
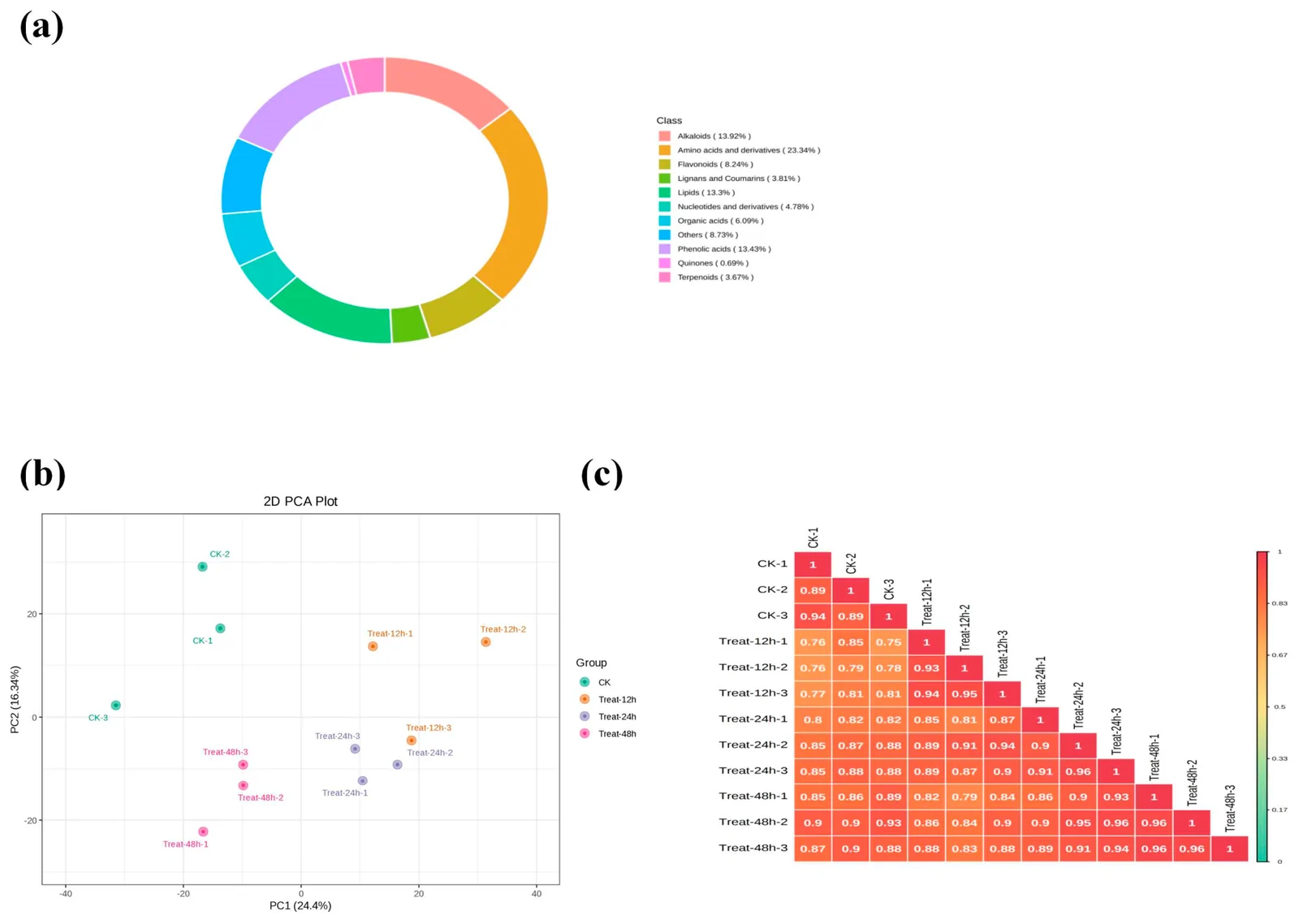
Metabolite classification and sample reproducibility analysis. (**a**) Classification of the identified metabolites according to chemical class. (**b**) Principal component analysis (PCA) of the 12 metabolomic samples. (**c**) Pearson correlation analysis among the 12 samples.

LC–MS-based metabolomic analysis was performed on samples from the CK, 12 h, 24 h, and 48 h groups, and a total of 1444 metabolites were identified. Based on their chemical classes, the identified metabolites were classified into 11 categories (Figure 8a), including amino acids and derivatives (23.34%), alkaloids (13.92%), phenolic acids (13.43%), lipids (13.30%), flavonoids (8.24%), organic acids (6.09%), nucleotides and derivatives (4.78%), lignans and coumarins (3.81%), terpenoids (3.67%), quinones (0.69%), and others (8.73%). Amino acids and derivatives represented the largest category among the identified metabolites.

Principal component analysis (PCA) was performed to evaluate the overall variation in metabolite profiles among the 12 samples (Figure 8b). PC1 and PC2 explained 24.40% and 16.34% of the total variation, respectively, accounting for 40.74% of the total variation. Samples from the four groups showed different distribution patterns in the PCA plot, while biological replicates within each group generally clustered together. Pearson correlation analysis further showed relatively high correlations among biological replicates within the same group (Figure 8c). Together, the PCA and correlation analyses indicated overall reproducibility of the metabolomic data, supporting their suitability for subsequent differential metabolite analysis.

#### 2.3.2. Identification of Differentially Accumulated Metabolites and KEGG Pathway Enrichment Analysis

**Figure 9:**
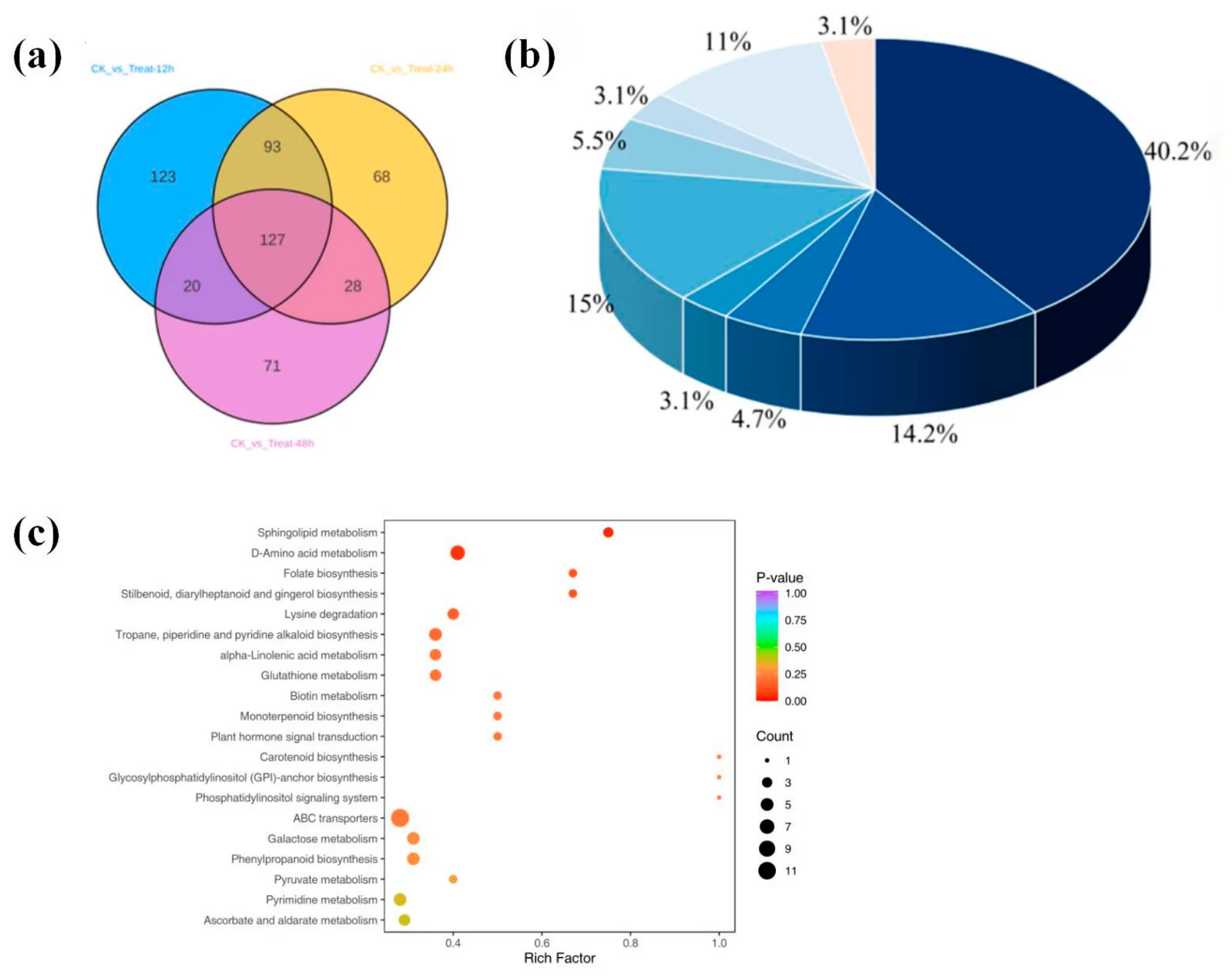
Differentially accumulated metabolites (DAMs) and KEGG pathway enrichment analysis following powdery mildew inoculation. (**a**) Venn diagram showing shared and comparison-specific DAMs among the CK vs. 12 h, CK vs. 24 h, and CK vs. 48 h comparisons. (**b**) Classification of the 127 DAMs shared among the three comparisons. Different colors represent different categories of shared differentially accumulated metabolites (DAMs), including amino acids and derivatives (40.2%), alkaloids (15.0%), phenolic acids (14.2%), lipids (11.0%), terpenoids (5.5%), flavonoids (4.7%), lignans and coumarins (3.1%), organic acids (3.1%), and other metabolites (3.1%). (**c**) KEGG pathway enrichment analysis of DAMs in the CK vs. 12 h comparison. Bubble size represents the number of DAMs assigned to each pathway, color represents the *p*-value, and the Rich Factor indicates the degree of pathway enrichment.

Differentially accumulated metabolites (DAMs) were identified using the criteria of variable importance in projection (VIP) > 1 and fold change (FC) ≥ 2 or ≤ 0.5. Based on these criteria, 363, 316, and 246 DAMs were identified in the CK vs. 12 h, CK vs. 24 h, and CK vs. 48 h comparisons, respectively (Figure 9a). Venn analysis identified 127 DAMs shared among all three comparisons. In addition, 123, 68, and 71 DAMs were specific to the CK vs. 12 h, CK vs. 24 h, and CK vs. 48 h comparisons, respectively. The 127 shared DAMs were classified into nine categories, with amino acids and derivatives representing the largest group (51 metabolites), followed by alkaloids (19), phenolic acids (18), lipids (14), terpenoids (7), flavonoids (6), lignans and coumarins (4), organic acids (4), and other metabolites (4) (Figure 9b).

KEGG pathway enrichment analysis was subsequently performed for the DAMs identified at different time points. At 12 h, the DAMs were assigned to 73 KEGG pathways, and pathways with an FDR < 0.05 were considered significantly enriched, and the top 20 significantly enriched pathways ranked by *p*-value were visualized using a bubble plot (Figure 9c and Supplementary Figure S9). Among these pathways, sphingolipid metabolism showed a high degree of enrichment, while ABC transporters contained the largest number of DAMs. Several pathways associated with amino acid, carbohydrate, lipid, and secondary metabolism were also represented, including D-amino acid metabolism, lysine degradation, galactose metabolism, pyruvate metabolism, pyrimidine metabolism, phenylpropanoid biosynthesis, and stilbenoid, diarylheptanoid, and gingerol biosynthesis. In addition, several pathways potentially associated with plant defense responses were enriched, including plant hormone signal transduction, α-linolenic acid metabolism, glutathione metabolism, ascorbate and aldarate metabolism, and carotenoid biosynthesis.

At 24 h, the DAMs were assigned to 55 KEGG pathways, and Pathways with an FDR < 0.05 were considered significantly enriched, and the top 20 significantly enriched pathways ranked by *p*-value were visualized (Supplementary Figure S9). Tropane, piperidine and pyridine alkaloid biosynthesis was among the significantly enriched pathways. Compared with the enrichment profile at 12 h, additional pathways associated with primary and specialized metabolism were represented at 24 h, including tyrosine metabolism, tryptophan metabolism, linoleic acid metabolism, flavone and flavonol biosynthesis, ether lipid metabolism, vitamin B6 metabolism, anthocyanin biosynthesis, and flavonoid biosynthesis.

At 48 h, the DAMs were assigned to 36 KEGG pathways (Supplementary Figure S9). Stilbenoid, diarylheptanoid and gingerol biosynthesis was among the significantly enriched pathways. Several additional metabolic pathways were represented at 48 h, including sulfur metabolism, arginine and proline metabolism, folate biosynthesis, and ubiquinone and other terpenoid-quinone biosynthesis. Overall, the KEGG enrichment profiles differed among the three post-inoculation time points, indicating temporal changes in metabolite accumulation and associated metabolic pathways during the response to powdery mildew.

### 2.4. Integrated Transcriptomic, Proteomic, and Metabolomic Analysis

**Figure 10:**
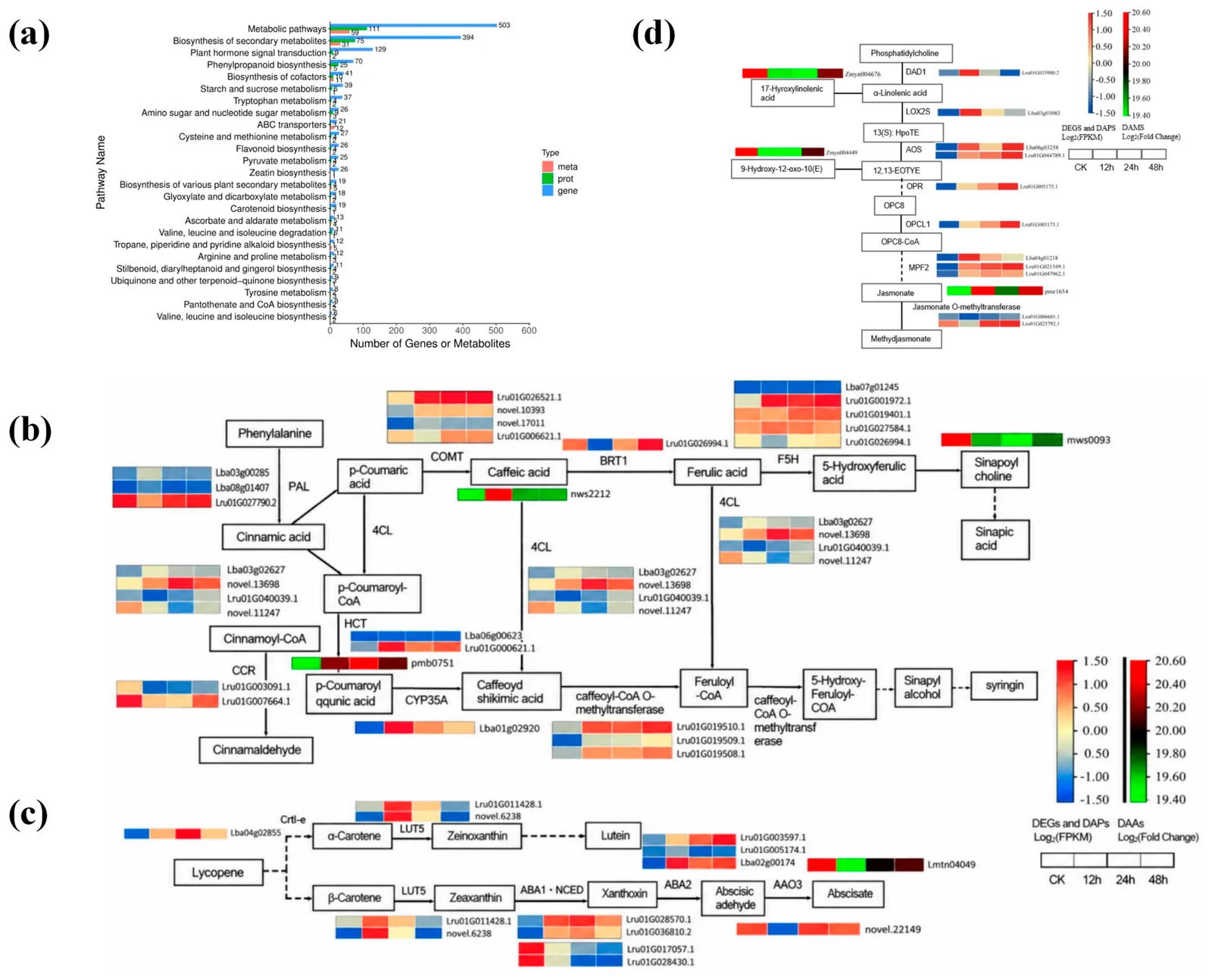
Integrated transcriptomic, proteomic, and metabolomic analysis of the response to powdery mildew inoculation. (**a**) Comparison of DEGs, DEPs, and DAMs assigned to shared KEGG pathways in the CK vs. 12 h comparison. (**b**) Integrated expression and accumulation patterns of genes, proteins, and metabolites involved in phenylpropanoid biosynthesis. (**c**) Integrated expression and accumulation patterns of molecular components involved in carotenoid biosynthesis and ABA metabolism. (**d**) Integrated expression and accumulation patterns of genes, proteins, and metabolites involved in α-linolenic acid metabolism and jasmonate biosynthesis.

KEGG pathways shared among the transcriptomic, proteomic, and metabolomic datasets were subjected to integrated analysis to characterize coordinated molecular responses to powdery mildew infection. At 12 h post-inoculation (hpi), 55 KEGG pathways were shared among the three omics datasets, and the top 25 pathways were compared based on the numbers of differentially expressed genes (DEGs), differentially expressed proteins (DEPs), and differentially accumulated metabolites (DAMs) assigned to each pathway (Figure 10a). Metabolic pathways contained the largest number of DEGs, whereas biosynthesis of secondary metabolites contained 394 DEGs, 75 DEPs, and 31 DAMs. Other pathways represented across the three omics datasets included plant hormone signal transduction, phenylpropanoid biosynthesis, carotenoid biosynthesis, flavonoid biosynthesis, and starch and sucrose metabolism.

At 24 hpi, 45 KEGG pathways were shared among the three omics datasets. Metabolic pathways contained large numbers of differential components, including 825 DEGs, 173 DEPs, and 41 DAMs. Changes were also observed in phenylpropanoid biosynthesis, carbon metabolism, starch and sucrose metabolism, and pyruvate metabolism. At 48 hpi, 24 KEGG pathways were shared among the three omics datasets. Metabolic pathways again contained large numbers of differential components, including 918 DEGs, 83 DEPs, and two DAMs. Plant hormone signal transduction contained 202 DEGs, four DEPs, and one DAM, while ubiquinone and other terpenoid-quinone biosynthesis and nicotinate and nicotinamide metabolism were also represented. Overall, the numbers of DEGs, DEPs, and DAMs assigned to individual pathways differed considerably, indicating that changes at the transcript, protein, and metabolite levels did not exhibit a simple one-to-one correspondence.

To further characterize the coordinated changes among the three molecular levels, representative defense-related metabolic pathways were examined. In the phenylpropanoid biosynthesis pathway, phenylalanine serves as an important precursor for the biosynthesis of numerous specialized metabolites (Figure 10b). During the conversion of phenylalanine to cinnamic acid and downstream derivatives, several genes and proteins associated with phenylalanine ammonia-lyase (PAL), 4-coumarate-CoA ligase (4CL), and cinnamoyl-CoA reductase (CCR) showed marked temporal changes. The *PAL* gene *Lru01G277092* showed decreased transcript abundance after inoculation, reaching its lowest level at 12 hpi and subsequently recovering. In contrast, the corresponding PAL proteins Lba03g00285 and Lba08g01407 showed increased abundance at 12 hpi followed by a subsequent decline, indicating discordant temporal patterns between transcript and protein abundance. Similar differences between transcript and protein abundance were also observed for components associated with 4CL and CCR.

Downstream of cinnamic acid, the phenylpropanoid pathway branched into multiple metabolic routes. Components associated with *COMT*, *BRT1*, and *F5H* showed dynamic expression patterns in branches leading to ferulic acid- and sinapic acid-related metabolites. Changes were also observed in metabolites and molecular components associated with *p*-coumaroyl shikimate and caffeoyl-CoA metabolism. These coordinated changes suggest extensive remodeling of phenylpropanoid metabolism following powdery mildew inoculation, potentially affecting the biosynthesis of lignin-related and other phenolic compounds. Lignin content, lignin deposition, and cell-wall composition were not directly measured, so these profiles do not establish increased lignification or cell-wall reinforcement.

Carotenoid metabolism was also affected following powdery mildew inoculation (Figure 10c). Starting from lycopene, the pathway diverged into α- and β-carotene-derived branches associated with xanthophyll and abscisic acid (ABA) biosynthesis. Metabolites associated with the lutein branch were not detected in the integrated dataset, whereas several genes associated with ABA biosynthesis, including *ABA1*, *ABA2*, *NCED*, and *AAO3*, showed temporal changes in expression. In the broad-range metabolomic dataset, the relative LC–MS/MS signal annotated as ABA also showed temporal changes following inoculation. Because ABA was not independently validated using an authentic standard in a targeted MRM assay, this observation should be interpreted as a relative change in the putatively annotated metabolite signal rather than a definitive quantitative change in ABA concentration.

The α-linolenic acid metabolism pathway also showed pronounced changes following powdery mildew inoculation (Figure 10d). Transcriptomic analysis identified DEGs encoding key enzymes involved in this pathway, including lipoxygenase (LOX), allene oxide synthase (AOS), and 12-oxophytodienoate reductase (OPR), with distinct temporal expression patterns observed among individual genes. Proteomic analysis further identified DEPs associated with the pathway, including LOX2S, AOS, and MPF2, whose abundance changed dynamically following inoculation. Metabolomic analysis identified DAMs associated with α-linolenic acid metabolism, including jasmonate-related metabolites. In the broad-range metabolomic dataset, the relative LC–MS/MS signal annotated as JA increased following inoculation and was highest at 12 hpi. Because JA was not independently validated using an authentic standard and targeted MRM analysis, this pattern represents a relative change in a putatively annotated metabolite signal rather than a validated change in JA concentration. Together with the transcriptomic and proteomic changes observed in the α-linolenic acid pathway, these data suggest that JA-associated metabolism may be involved in the response to powdery mildew infection.

### 2.5. Construction of a Gene-Editing Vector for LbMLO6 and Genetic Transformation Using CRISPR/Cas9 Technology

#### 2.5.1. Bioinformatic Characterization and Expression Analysis of *LbMLO6*

Integrated analysis of the transcriptomic and proteomic datasets identified an *MLO* family gene, *Lru01G016286.1* (“01” refers to isolate Lr01, not chromosome 1), that showed increased expression following powdery mildew inoculation. Sequence analysis identified this gene as *LbMLO6*, which was subsequently selected for further characterization.

**Figure 11:**
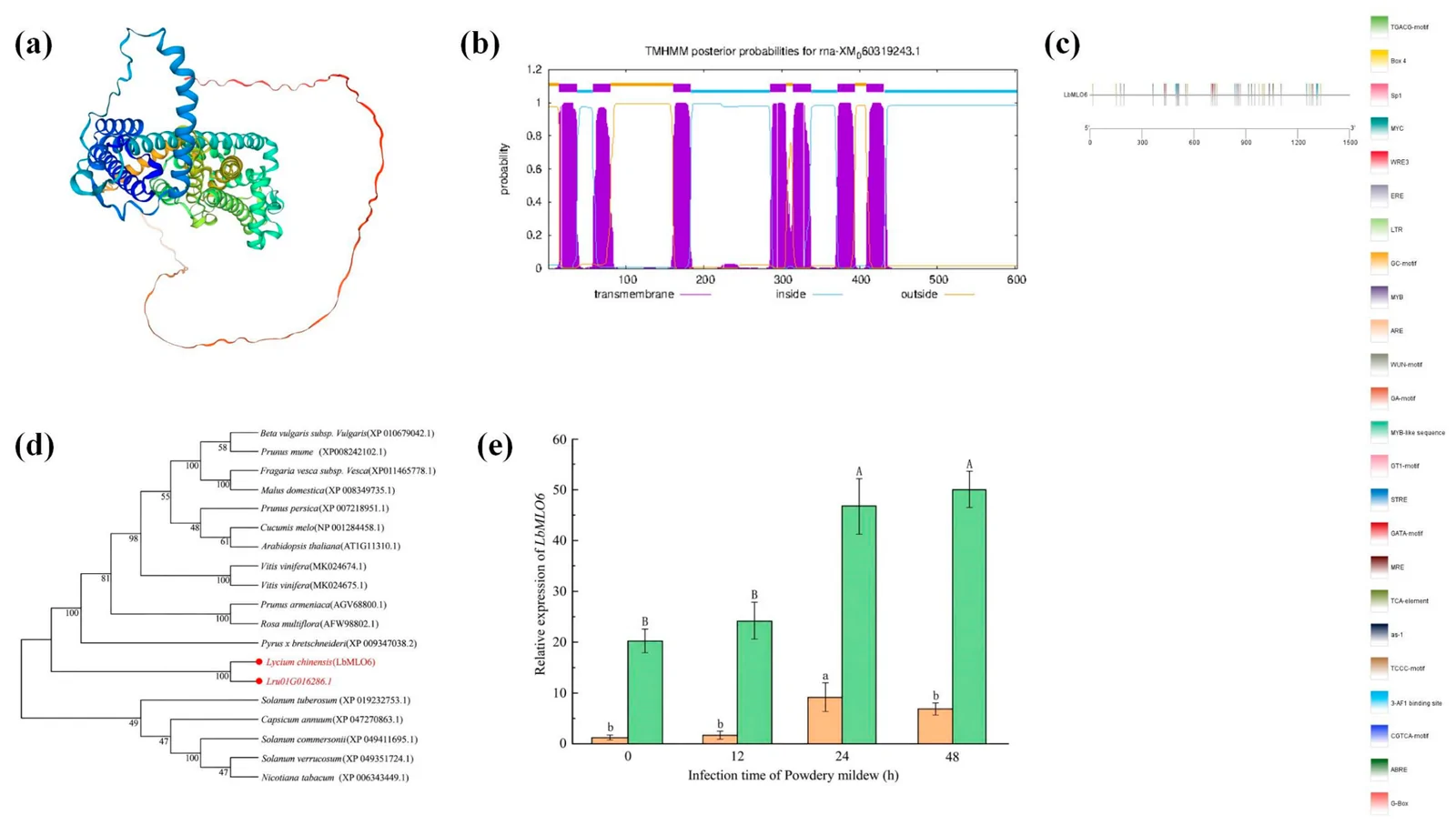
Bioinformatic characterization and expression analysis of *LbMLO6*. (**a**) Predicted tertiary structure of the LbMLO6 protein. (**b**) Predicted transmembrane topology of LbMLO6 (*Lru01G016286.1*, corresponding to XM_019243.1). (**c**) Distribution of putative cis-acting regulatory elements in the *LbMLO6* promoter. (**d**) Phylogenetic analysis of LbMLO6 and MLO proteins from other plant species. (**e**) Comparison of *LbMLO6* expression patterns determined by RNA-seq and RT-qPCR. Green bars represent RNA-seq data, and orange bars represent RT-qPCR data. Different lowercase letters indicate significant differences among infection time points in the qPCR data, whereas different uppercase letters indicate significant differences among infection time points in the transcriptomic data (*p* < 0.05).

The LbMLO6 protein consists of 603 amino acids, with a predicted molecular weight of 68.85 kDa and a theoretical isoelectric point (pI) of 8.89. The predicted instability index was 50.22, suggesting that LbMLO6 is an unstable protein. Its aliphatic index was 90.96, and the grand average of hydropathicity (GRAVY) was −0.073. Chromosomal mapping indicated that *LbMLO6* is located at approximately 13.75 Mb on chromosome 8. Subcellular localization prediction suggested that LbMLO6 is localized to the plasma membrane. Secondary-structure prediction indicated that the protein consists predominantly of α-helices and random coils, accounting for 44.11% and 50.41% of the sequence, respectively, while extended strands accounted for 5.47%. The predicted tertiary structure of LbMLO6 is shown in Figure 11a.

Multiple sequence alignment (Supplementary Figure S13) and transmembrane topology prediction showed that LbMLO6 contains seven predicted transmembrane helices, a characteristic structural feature of *MLO* family proteins (Figure 11b). The predicted topology placed the N-terminus on the extracellular side and the C-terminus on the cytoplasmic side of the membrane. These structural characteristics support the classification of *LbMLO6* as an *MLO* family membrane protein.

To investigate the potential transcriptional regulation of *LbMLO6*, cis-acting regulatory elements were analyzed within the approximately 1.5 kb promoter region upstream of the transcription start site (Figure 11c). The promoter contained multiple putative cis-acting elements associated with hormone, stress, and light responses. Hormone-responsive elements included ABRE, TGACG-motif, CGTCA-motif, TCA-element, and ERE, which are associated with responses to abscisic acid, methyl jasmonate, salicylic acid, and ethylene. Stress-related elements included MYB- and MYC-binding sites, WUN-motif, LTR, and STRE, while light-responsive elements included Box 4, G-Box, and GT1-motif. The presence of these putative cis-regulatory elements suggests that *LbMLO6* expression may respond to multiple hormonal and environmental signals.

Phylogenetic analysis was performed using LbMLO6 and MLO proteins from other plant species (Figure 11d). LbMLO6 clustered with MLO proteins from other dicotyledonous species, including *Pyrus × bretschneideri.* Together with its sequence characteristics and predicted seven-transmembrane topology, the phylogenetic relationship further supports the classification of LbMLO6 as a member of the plant MLO protein family. Representative reference proteins for established MLO clades were not included in this analysis. Consequently, the tree did not resolve the clade assignment of LbMLO6 and does not establish whether it belongs to or falls outside clade V.

The expression pattern of *LbMLO6* was further examined by RT-qPCR and compared with the RNA-seq data (Figure 11e). RNA-seq analysis showed that *LbMLO6* transcript abundance remained relatively stable between CK and 12 hpi, followed by a marked increase at 24 hpi and a further increase to the highest level at 48 hpi. RT-qPCR analysis also showed increased *LbMLO6* expression following inoculation, with expression increasing markedly at 24 hpi and remaining at a relatively high level at 48 hpi, although it decreased slightly compared with that at 24 hpi. Thus, although the detailed temporal patterns differed between RNA-seq and RT-qPCR, both datasets indicated that the marked induction of *LbMLO6* occurred mainly from 24 hpi onward.

#### 2.5.2. Construction of the *LbMLO6* Gene-Editing Vector and Agrobacterium-Mediated Genetic Transformation

**Figure 12:**
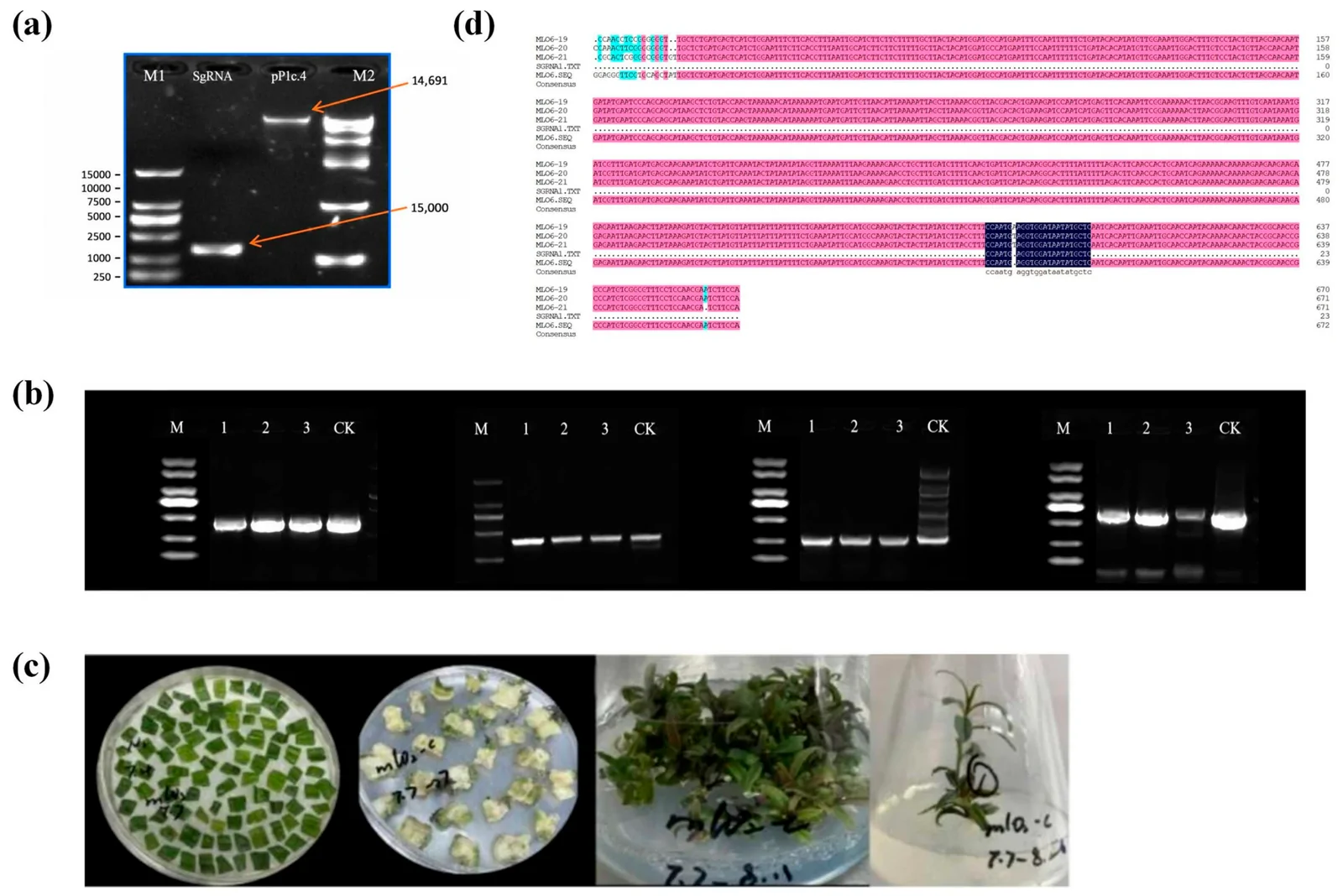
Construction of the *LbMLO6* CRISPR/Cas9 gene-editing vector and generation of edited lines. (**a**) PCR amplification of the sgRNA-containing fragment and linearization of the CRISPR/Cas9 vector. (**b**) Molecular verification of candidate recombinant clones by PCR and restriction enzyme digestion. M, DNA marker; lanes 1–3, candidate clones; CK, negative control. (**c**) Representative stages of Agrobacterium-mediated genetic transformation and plant regeneration, including co-cultivation, callus formation, shoot regeneration, and rooting. (**d**) Sequence alignment of the *LbMLO6* target region in representative CRISPR/Cas9-edited lines MLO6-19, MLO6-20, and MLO6-21.

The sgRNA-containing fragment was amplified using a high-fidelity DNA polymerase and subsequently ligated into the linearized CRISPR/Cas9 vector. As shown in Figure 12a, the amplified sgRNA-containing fragment was approximately 350 bp, while the linearized vector was approximately 14.7 kb, consistent with the expected sizes. Recombinant colonies were screened by colony PCR and restriction enzyme digestion (Figure 12b). Plasmids from verified positive colonies were subsequently introduced into *Agrobacterium tumefaciens* strain GV3103 for genetic transformation.

Genetic transformation was performed through preculture, co-cultivation, selection, shoot regeneration, and rooting (Figure 12c). A total of 24 candidate lines were obtained and subjected to molecular identification. PCR amplification was performed to verify the presence of the CRISPR/Cas9 construct, and the *LbMLO6* target regions were subsequently amplified and sequenced to determine the editing patterns. The sgRNA1 and sgRNA2 target regions were amplified and sequenced to evaluate editing at both target sites. Sequence analysis showed that mutations were detected at the sgRNA1 target site in 21 of the 24 candidate lines, corresponding to an editing efficiency of 87.5%. In contrast, no mutations were detected at the sgRNA2 target site among the examined lines. Thus, detectable editing in the regenerated plants was restricted to the sgRNA1 target site.

Different types of mutations, including nucleotide insertions and deletions, were identified among the 21 edited lines (Figure 12d and Supplementary Figures S14–S17). Lines 1 and 22 exhibited relatively large deletions; lines 2–13 and 19–21 carried homozygous mutations involving A/T insertions; lines 15, 17, and 24 carried a T deletion; and line 16 carried an AC insertion. Among the 21 successfully edited lines, 15 were identified as containing homozygous mutations in the sampled leaf tissues, accounting for 71.4% of the edited lines (Table 1). These results confirmed successful CRISPR/Cas9-mediated editing of *LbMLO6* and the generation of multiple *Lbmlo6* edited lines with distinct mutation patterns.

#### 2.5.3. Qualitative Assessment of Powdery Mildew Infection and Leaf Morphological Traits in *Lbmlo6* Edited Lines

**Figure 13:**
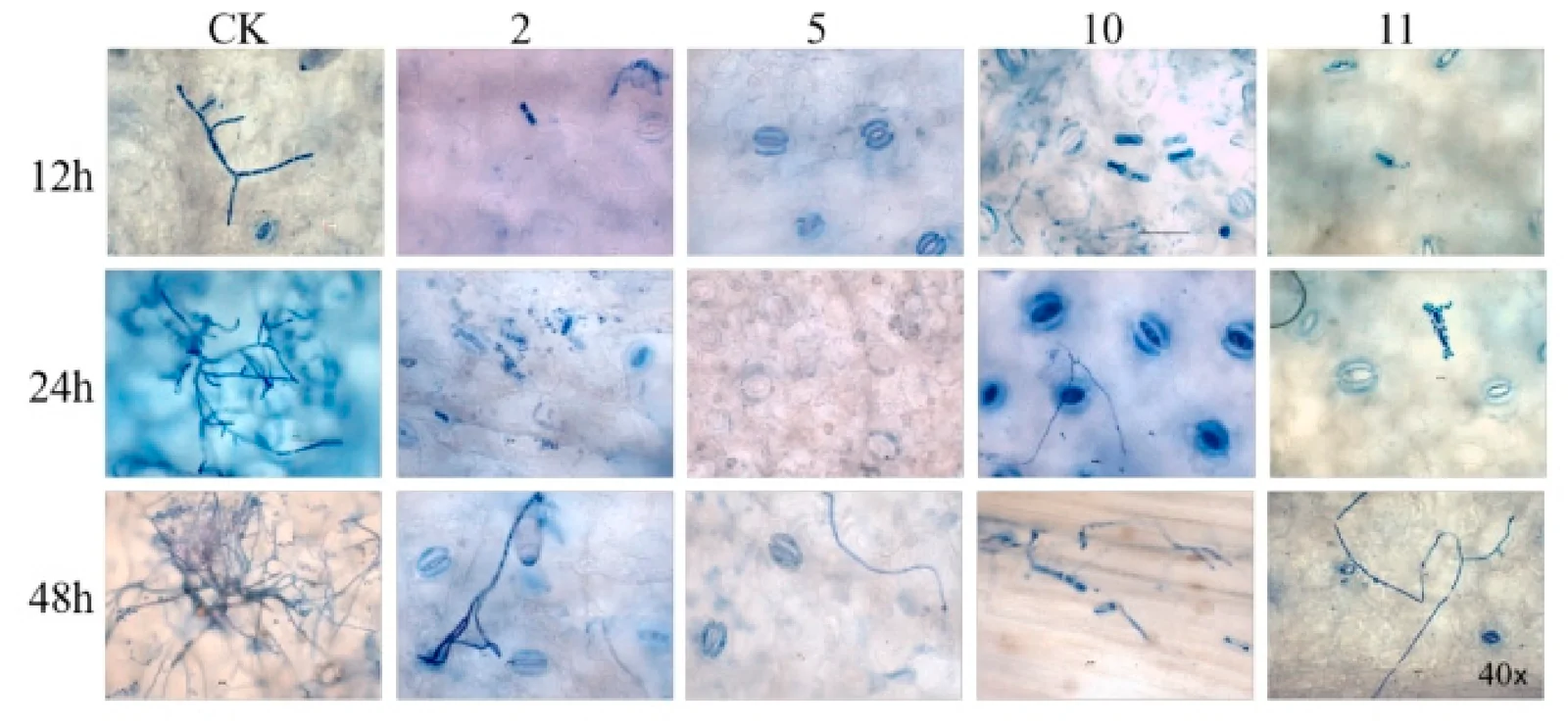
Trypan blue staining of powdery mildew infection in regenerated non-edited CK plants and representative Lbmlo6 edited lines. CK plants underwent the same tissue-culture and regeneration procedures but showed no detectable editing at the *LbMLO6* target sites. Microscopic observations of CK and edited lines 2, 5, 10, and 11 were performed at 12, 24, and 48 h post-inoculation (hpi). Images were obtained at 40× magnification.

To evaluate the response of *Lbmlo6* edited lines to powdery mildew infection, representative edited lines were inoculated with the powdery mildew pathogen and examined by trypan blue staining at 12, 24, and 48 h post-inoculation (hpi) (Figure 13). Regenerated Ningqi-1 plants that underwent the same tissue-culture and regeneration procedures but showed no detectable editing at the *LbMLO6* target sites were used as the CK control. Compared with the regenerated non-edited CK plants, several edited lines showed less extensive trypan-blue-stained fungal structures at 12 and 24 hpi, although fungal development varied among individual edited lines. By 48 hpi, fungal development in the edited lines appeared comparable to that in CK. Fungal biomass and disease severity were not quantitatively measured, and observations did not extend beyond 48 hpi. An empty-vector transformed control was not included. These observations therefore document qualitative differences in early fungal development but do not establish sustained resistance or distinguish effects of *LbMLO6* editing from those associated with transformation or the vector background.

Leaf morphological traits were further evaluated in regenerated non-edited CK plants and edited lines 2, 5, 10, and 11 (Table 2). The measured traits included leaf length, maximum width, blade thickness, leaf area, and length-to-width ratio. Values represent mean ± standard error (SE). Different lowercase letters indicate significant differences among groups according to one-way analysis of variance (ANOVA), followed by Tukey’s HSD (*p* < 0.05). Leaf length did not differ significantly between the edited lines and CK. In contrast, several edited lines showed reduced maximum leaf width, with lines 2 and 5 exhibiting particularly pronounced reductions. Line 2 showed a lower mean blade thickness and reduced leaf area compared with CK; however, the relatively large SE observed for blade thickness warrants cautious interpretation of this trait. The length-to-width ratio tended to be higher in the edited lines, particularly in lines 2 and 5, indicating a tendency toward a narrower leaf phenotype in some *Lbmlo6* edited lines. These results suggest that *LbMLO6* editing may be associated with changes in leaf morphology; however, the magnitude of these changes varied among individual edited lines. The present comparisons do not distinguish effects of *LbMLO6* disruption from regeneration-associated variation or unintended editing effects.

## 3. Discussion

Because powdery mildew fungi are obligate biotrophs, a standardized conidial suspension prepared from freshly infected leaves was used for inoculation. This approach improved consistency among biological replicates while retaining a biologically relevant infection route, thereby providing a reproducible basis for subsequent multi-omics comparisons.

Transcriptomic analysis revealed broad transcriptional reprogramming in wolfberry leaves following powdery mildew infection, with defense-related responses converging on Ca^2+^-associated signaling, WRKY-mediated transcriptional regulation, ROS-associated responses, hormone signaling, and phenylpropanoid metabolism. The coordinated changes in genes encoding CNGCs, CaM/CML proteins, WRKY transcription factors, and *PR-1* suggest that pathogen perception is accompanied by remodeling of calcium-dependent signaling and downstream defense regulation. These transcriptional responses provide a molecular framework for the protein- and metabolite-level changes observed in the subsequent multi-omics analyses.

Plant hormone signaling also appears to be an important component of the wolfberry response to powdery mildew infection. Transcriptomic analysis revealed substantial changes in genes associated with ethylene (ET), jasmonic acid (JA), salicylic acid (SA), and abscisic acid (ABA) signaling. In particular, SA-associated components, including *NPR1*, *TGA*, and *PR-1*, exhibited pronounced transcriptional responses, with strong induction of *PR-1* at the later stages of infection. The broad-range metabolomic analysis further detected relative LC–MS/MS signals annotated as JA and ABA that showed temporal changes following inoculation. Together with the transcriptomic responses of hormone-associated genes, these metabolomic signals suggest that multiple hormone-related processes may be associated with the response to powdery mildew infection. The present data do not establish antagonistic, synergistic, or causal interactions among these hormone pathways. Targeted hormone quantification and genetic or pharmacological perturbation are needed to determine the nature of their interactions.

Previous studies have shown that SA-associated signaling and NPR1 activity can influence the expression of genes involved in phenylpropanoid and flavonoid metabolism [34]. Comparative transcriptomic studies in other plant–pathogen systems have similarly demonstrated close associations among hormone signaling, lignin biosynthesis, peroxidase activity, and plant defense [35]. Consistent with these findings, the proteomic analysis performed in the present study revealed changes in numerous defense- and cell-wall-associated proteins, including peroxidases, chitin-associated proteins, pectin-related enzymes, and glycoside hydrolases. KEGG and protein-domain enrichment analyses further highlighted pathways and protein families associated with phenylpropanoid metabolism, MAPK signaling, oxidative reactions, carbohydrate metabolism, and cell-wall modification. These findings suggest that the response of wolfberry to powdery mildew involves both signaling-mediated regulation and biochemical processes associated with defense and cell-wall remodeling.

To move beyond pathway-level description, we examined two candidate molecules that are co-supported by the transcriptomic, proteomic, and metabolomic layers and that lie at the core of the phenylpropanoid–cell-wall response. The first is *Lru01G035232.1*, annotated as a lignin-forming anionic class III peroxidase and assigned to phenylpropanoid biosynthesis (ko00940). Its transcript was consistently induced at 12, 24, and 48 h post-inoculation, and peroxidase abundance was independently altered in the proteomic profile, indicating convergence at both the RNA and protein levels. Class III peroxidases use apoplastic H_2_O_2_ to oxidize monolignols into radicals that polymerize into lignin and cross-link cell-wall proteins, thereby reinforcing the wall at infection sites [36]; knockdown of apoplastic class III peroxidases impairs the peroxidase-dependent oxidative burst and increases susceptibility to both fungal and bacterial pathogens [37]. The second candidate, *Lru01G023684.1*, is annotated as cinnamyl alcohol dehydrogenase 8 (CAD8; ko00940) and was also induced at all three time points. CAD8 catalyzes the final, committed step of monolignol biosynthesis, reducing coniferyl aldehyde to coniferyl alcohol, the guaiacyl monolignol whose relative accumulation was detected in the present metabolomic analysis. Pathogen-inducible CADs are established contributors to defense-related lignification and disease resistance [38,39,40]. Together, these observations suggest a possible link between CAD8-associated monolignol biosynthesis and peroxidase-associated cell-wall modification. The convergence of the multi-omics evidence identifies *Lru01G035232.1* and *Lru01G023684.1* as priority candidates for testing this proposed connection.

Metabolomic analysis further revealed substantial changes in specialized metabolism following powdery mildew infection. Among the commonly altered metabolites, several compounds were associated with phenylpropanoid metabolism, including 5-*O*-*p*-coumaroylquinic acid, trans-5-*O-p*-coumaroylshikimic acid, and coniferyl alcohol. Coniferyl alcohol is an important monolignol precursor involved in lignin biosynthesis, whereas coumaroyl-derived intermediates participate in upstream phenylpropanoid reactions. Integrated transcriptomic, proteomic, and metabolomic analyses therefore suggest extensive remodeling of phenylpropanoid metabolism during powdery mildew infection. The changes in monolignol-related metabolites and cell-wall-associated proteins suggest possible involvement of lignin-related metabolism, while their contribution to cell-wall reinforcement remains to be established.

The integrated multi-omics analysis also revealed that changes at the transcript, protein, and metabolite levels were not always directly correlated. For example, in the phenylpropanoid pathway, several genes and their corresponding proteins exhibited different temporal patterns, suggesting that transcriptional regulation alone does not fully determine protein accumulation. Post-transcriptional regulation, protein turnover, and differences in metabolic flux may contribute to these discrepancies. Similarly, metabolites associated with hormone responses were primarily assigned to hormone biosynthetic or precursor-related metabolic pathways rather than directly to the KEGG plant hormone signal transduction pathway. This distinction reflects the different molecular entities measured by transcriptomics, proteomics, and metabolomics and highlights the importance of integrating multiple molecular levels when interpreting plant responses to pathogen infection.

Specific *MLO* family members are well-established susceptibility factors for powdery mildew, and their loss of function can enhance powdery mildew resistance in a range of plant species [41,42,43]. Based on its infection-responsive expression pattern and the integrated multi-omics evidence, *LbMLO6* was selected as a candidate susceptibility-associated gene for further functional investigation. Bioinformatic analysis indicated that LbMLO6 possesses seven predicted transmembrane helices, consistent with the typical structural characteristics of *MLO* family proteins. The relationship of LbMLO6 to established clade-V susceptibility factors remains uncertain. Its infection-responsive expression and the transient, qualitative reduction in fungal development observed in edited lines suggest possible involvement in the infection process, but do not establish a susceptibility function or its mechanism. A phylogeny incorporating established clade references, together with quantitative disease assessment and genetic complementation, is needed to clarify its evolutionary placement and functional contribution. In addition, the promoter region of LbMLO6 contains multiple predicted cis-regulatory elements associated with hormone and stress responses, suggesting that its transcription may be regulated by multiple environmental and endogenous signals.

The infection-responsive behavior of *LbMLO6* and the SA-associated transcriptional signature described above are not mutually exclusive branches of the defense response, and existing literature supports a potential regulatory interface between MLO proteins and salicylic acid (SA)-mediated defense. In the present transcriptome, genes encoding *NPR1*, *TGA* transcription factors, and *PR-1* showed pronounced and temporally distinct induction—peaking at 24–48 h post-inoculation (Figure 3d), with PR-1 most strongly induced at 48 h—consistent with an SA-associated transcriptional response during infection. In *Arabidopsis*, *AtMLO2* is transcriptionally induced by SA and is required for execution of systemic acquired resistance, positioning MLO2 as a downstream component of the SA-dependent defense program rather than merely a penetration-susceptibility factor [44]. Consistent with an SA–MLO link, *Atmlo2*-mediated penetration resistance has in some genetic backgrounds been reported to require intact SA signaling [45]; however, the classic *mlo*-based resistance of barley and *Arabidopsis* operates largely independently of SA, NPR1, and other canonical defense hormones, relying instead on PEN-dependent papilla formation [46]; thus, loss of MLO function per se does not necessarily engage SA signaling. In the present study, the SA-associated transcriptional signature co-occurred with infection-responsive *LbMLO6* expression, but this association does not establish a regulatory relationship or its direction. Manipulating the SA pathway in *Lbmlo6* edited and control plants, together with *PR-1* expression analysis, could help test the proposed connection.

CRISPR/Cas9-mediated editing of *LbMLO6* generated multiple mutant lines with different mutation types. The contrasting editing outcomes observed for the two target sites suggest substantial target-dependent variation in editing efficiency. The absence of detectable mutations at sgRNA2 may reflect differences in guide RNA activity, local sequence context, chromatin accessibility, or other factors affecting Cas9-mediated cleavage. Although the precise reason for the lack of detectable sgRNA2 editing remains unclear, this result highlights the importance of evaluating multiple target sites when designing genome-editing strategies in *L. barbarum*.

Several of the detected insertions and deletions are expected to alter the coding sequence of *LbMLO6*. In particular, one- or two-nucleotide insertions or deletions within a coding region may cause frameshift mutations and potentially introduce premature termination codons, thereby potentially affecting LbMLO6 protein function. The early differences in fungal development suggest possible involvement of LbMLO6 in infection. Establishing a causal contribution will require quantitative disease assessment over a longer infection period, appropriate transformation controls, and genetic complementation in independent, genetically characterized edited lines.

MLO proteins may also have biological functions beyond disease susceptibility, and loss-of-function *mlo* mutants in several species are well documented to incur pleiotropic growth penalties—the so-called *mlo* trade-off effect. In barley, classic *mlo* alleles confer durable, broad-spectrum powdery mildew resistance but are accompanied by spontaneous mesophyll cell death, premature leaf senescence, necrotic leaf spotting, and a measurable yield penalty that is widely interpreted as a pleiotropic cost of *mlo*-mediated resistance [47,48]. By contrast, loss of function of the tomato *SlMlo1* gene (*ol-2)* provides full powdery mildew resistance without obvious growth or fruit-development penalties, and edited *slmlo1* lines lack the early-senescence chlorosis seen in barley and *Arabidopsis mlo* mutants [49,50]. In grapevine, CRISPR/Cas9 disruption of *VvMLO6* and *VvMLO7* confers immunity to *Erysiphe necator* but is associated with a decline in photosynthesis-related processes, transcriptional reprogramming of carbohydrate metabolism, and overaccumulation of stilbenes together with enhanced oxidative-scavenging potential—metabolic shifts that have been proposed to buffer the mutants against detrimental pleiotropic effects [21]. These contrasting examples illustrate that the severity and nature of mlo-associated pleiotropy are highly species- and allele-dependent. In the present study, leaf morphological traits varied among the *Lbmlo6* edited lines, with some lines showing reduced leaf width and increased length-to-width ratios (a tendency toward narrower leaves), whereas the magnitude of these changes differed among individual lines. The leaf-shape differences warrant investigation of possible developmental effects of *LbMLO6* editing. Independent genetically characterized lines, complementation, and longer-term growth, yield, and field assessments would help clarify the origin and agronomic significance of these morphological differences.

Taken together, the multi-omics data support a preliminary model of the wolfberry response to powdery mildew infection. Pathogen infection is associated with changes in Ca^2+^-dependent signaling, WRKY-mediated transcriptional regulation, ROS-associated responses, and multiple hormone signaling pathways. These signaling events are accompanied by changes in defense-related proteins and extensive remodeling of phenylpropanoid and other secondary metabolic pathways. Changes in lignin-related metabolic intermediates and cell-wall-associated proteins suggest possible cell-wall remodeling during infection. Transcriptomic changes in multiple hormone-associated pathways, together with relative metabolomic signals annotated as JA and ABA, suggest that hormone-related processes are dynamically associated with the infection response.

Nevertheless, the regulatory framework proposed here is based primarily on multi-omics associations and preliminary genetic evidence. The relationships among transcriptional regulation, protein accumulation, metabolite changes, and disease resistance therefore require further experimental validation.

## 4. Materials and Methods

### 4.1. Experimental Materials

The material used in this experiment consisted of Ningqi-1 plants established at the Goji Berry Research Institute of the Ningxia Academy of Agricultural and Forestry Sciences. These plants were subjected to conventional cultivation and management; the goji berry plants used for inoculation with the powdery mildew pathogen to determine physiological indicators, as well as those sent for transcriptomic, proteomic and metabolomic analyses, were cuttings of Ningqi-1. Powdery mildew conidia used for inoculation were collected from naturally infected leaves of Tianjing-3, a wolfberry cultivar highly susceptible to powdery mildew. Because molecular identification of the pathogen was not performed in the present study, the inoculum is referred to here as the powdery mildew pathogen. The materials used for genetic transformation were seeds from the progeny of Ningqi-1; after sterilization, these were sown on MS solid medium to produce seedlings.

Both the Ningqi-1 cuttings and the seed-grown seedlings were cultured in the Plant Tissue Culture Laboratory at Northern Minzu University under the following conditions: 25 °C, 12 h light/12 h dark photoperiod, with a light intensity of 4000 lux.

The CRISPR/Cas9 vector used for gene editing in this experiment was pP1C.4, purchased from Jirui Biotechnology (Nanjing, China); the *E. coli* strain used for vector construction was GT115 competent cells, purchased from Zeye Biotechnology Co., Ltd. (Shanghai, China); and the *Agrobacterium tumefaciens* used for genetic transformation was GV3101, purchased from Biyuntian Biotechnology Co., Ltd. (Shanghai, China).

### 4.2. Experimental Methods

#### 4.2.1. Transcriptome Sequencing and Analysis

*Lycium barbarum* L. cv. Ningqi-1 rooted seedlings with consistent growth performance at 25 days after rooting of cuttings were selected for powdery mildew inoculation experiments. Each treatment was performed with three independent biological replicates. For each biological replicate, the 4th–6th functional leaves of 10 randomly selected plants were collected and pooled for sampling. The spore suspension of the powdery mildew pathogen was adjusted to a final concentration of 2 × 10^6^ spores/mL. Spore germination was examined prior to inoculation; only spore suspensions with a germination rate higher than 90% were selected. The suspension was uniformly sprayed onto the plants from a height of 30 cm above the canopy, with 3 mL of spore suspension applied to each plant. Sterile water treatment was used as the control.

After inoculation, the 4th–6th functional leaves were collected at 0, 12, 24, and 48 hpi. The functional leaves from each treatment were pooled, with 0.6 g of tissue collected per sample for transcriptomic analysis, resulting in a total of 12 transcriptome samples. Proteomic and metabolomic analyses and multi-omics data validation were performed using the same sampling strategy as that used for transcriptomic analysis. All samples were immediately frozen in liquid nitrogen, stored on dry ice, and transported to Wuhan Metware Biotechnology Co., Ltd. (Wuhan, China) for transcriptome, proteome, and metabolome analyses.

#### 4.2.2. Validation by Real-Time Quantitative PCR (qPCR)

Using the Plant Polysaccharide and Polyphenol Total RNA Extraction Kit (Tiangen Biotech, Beijing, China), total RNA was extracted from *Lycium barbarum* leaves at four time points post-inoculation. The RNA was then reverse-transcribed into cDNA using the FastKing RT Kit (Tiangen Biotech, Beijing, China); detailed procedures are provided in the kit manual.

Two internal reference genes (*Actin1* and *GAPDH*) and 12 genes showing high fold changes in expression (*Lru01G017344.1*, *Lru01G041208.1*, *Lru01G049867.1*, *Lru01G007387.1*, *Lru01G040980.1*, *Lru01G039198.1*, *Lru01G019510.1*, *Lru01G011568.1*, *Lru01G048735.1*, *Lru01G026673.1*, *Lru01G039592.1*, *novel.5673*; among these, *novel.5673* was annotated as *CML49* based on SwissProt, but has not yet been assigned an Lru0lG-format gene model identifier). were selected for qPCR validation. Primers were designed using Primer 5.0; the primer sequences are shown in Appendix A Table A1. qPCR validation was performed in accordance with the experimental procedures for the SGExcel FastSYBR Mixture kit (Sangon Biotech, Shanghai, China). Relative expression levels were calculated using the 2^−ΔΔCT^ method, and a composite plot of relative gene expression was generated with transcriptomic data on the *x*-axis and qPCR data on the *y*-axis.

#### 4.2.3. Broad-Range Metabolomic Profiling and Analysis

Extraction, detection, and analysis of metabolites: Twelve goji berry leaf samples were vacuum freeze-dried and ground into a fine powder. A 50 mg aliquot of each powdered sample was used for metabolite extraction, and 1200 μL of 70% (*v*/*v*) methanol extraction solution, pre-cooled to −20 °C, was added. The mixture was vortexed six times at 30 min intervals. The mixture was centrifuged, and the supernatant was collected. Finally, the supernatant was filtered through a 0.22 μm filter membrane into a sample vial for liquid chromatography–mass spectrometry (LC–MS/MS) analysis. Metabolite detection was performed using a UPLC–ESI–MS/MS system. Multiple reaction monitoring (MRM) was used for metabolite detection and relative quantification. Characteristic ions for individual metabolites were selected using a triple-quadrupole mass spectrometry system, and the corresponding ion signals were recorded. For each metabolite, Q1 represented the precursor (parent) ion selected after electrospray ionization, whereas Q3 represented a characteristic fragment ion. According to the ionization model used for individual metabolites, [M + H]^+^ represented positively ionized species and [M − H]^−^ represented negatively ionized species. Chromatographic peaks were integrated and corrected using MultiQuant software (version 3.0; SCIEX, Framingham, MA, USA), and the integrated peak area was used to represent the relative abundance of each metabolite. Metabolite annotation was performed by comparison with an in-house metabolite database. The identification criteria included MS/MS fragment-ion and retention-time (RT) matching, whereas Q1, Q3, RT, declustering potential (DP), and collision energy (CE) were used as database-matching parameters for metabolites assigned to the corresponding identification level. Detailed UPLC–MS/MS sampling quantities and analytical conditions are provided in Appendix A Table A2. Definitions of Q1, Q3, ionization mode, the relative quantification procedure, and metabolite-identification criteria are summarized in Appendix A Table A3.

DDA-PASEF proteomics detection and analysis: Sample extraction was based on the TCA-acetone precipitation method described in previous studies, with some modifications, to extract proteins [51,52]. One hundred micrograms of the extracted protein was digested with trypsin overnight at 37 °C. The peptides were desalted using C18, freeze-dried under vacuum, resuspended to a concentration of 0.1 μg/μL, and stored at −20 °C for later use.

LC–MS/MS analysis: Sample separation was performed using a NanoElute system (Bruker Daltonik, Bremen, Germany) at a nanoliter flow rate. Mobile phase A consisted of 0.1% (*v*/*v*) formic acid in water, whereas mobile phase B consisted of 0.1% (*v*/*v*) formic acid in acetonitrile. Samples were loaded onto the analytical column via an autosampler for separation. Following chromatographic separation, mass spectrometry data were acquired using the dda-PASEF mode on a timsTOF Pro 2 mass spectrometer (Bruker Daltonics, Bremen, Germany). The metabolomic analysis was performed as a broad-range relative profiling analysis. Phytohormone-related metabolites, including signals annotated as JA and ABA, were not independently confirmed or absolutely quantified using authentic chemical standards in targeted MRM assays. Therefore, changes in these metabolites were interpreted as relative LC–MS/MS signal changes rather than definitive measurements of phytohormone concentrations.

To reduce the false positive rate caused by multiple comparisons, the Benjamini–Hochberg method was applied for false discovery rate (FDR) correction in all significance analyses. GO and KEGG enrichment analyses were considered statistically significant when the FDR value was <0.05. The screening of differentially expressed omics data simultaneously considered both the fold change (FC) and statistical significance level.

#### 4.2.4. Construction of a Gene-Editing Vector for *LbMLO6* and Genetic Transformation Using CRISPR/Cas9 Technology

Leaves from healthy Ningqi-1 (*Lycium barbarum* L.) plants were selected for RNA extraction and reverse transcribed into cDNA. Gene cloning was performed using the Full-Form Gold 2× TransStart^®^ KD Plus PCR SuperMix (TransGen Biotech, Beijing, China) with high-fidelity polymerase; the PCR reaction protocol is shown in Appendix A Table A4. The products were electrophoresed on a 1.5% agarose gel, the bands were excised and recovered, and the samples were sent to Shanghai Geneworks (Shanghai Generay Biotech, Shanghai, China) for sequencing. After verifying the sequence alignment, primers for the gene-editing target site were designed using CRISPR-P2.0 (http://crispr.hzau.edu.cn/CRISPR2/; accessed on 18 October 2026). The target site was selected, and gene-editing primers were designed in accordance with primer design principles, as shown in Appendix A Table A5. Based on the sequenced data, the relevant physicochemical properties, subcellular localization, and transmembrane domains of the protein were predicted (Appendix A Table A6 for websites and URLs), and MEGA was utilized to perform multiple sequence alignments with MLO proteins from other species with established functions and to construct a phylogenetic tree.

Using a high-fidelity DNA polymerase, with the pP1C.4 vector as a template and U6p.4-F, *SgRNA1-R* and *SgRNA2-R* as primers, PCR amplification was performed to obtain the SgRNA cloning frame. Following amplification, the PCR products were subjected to gel electrophoresis, and the target fragment was recovered. The pP1C.4 vector was double-digested with *EcoR* I and *Xba*I, and a recombinant vector was constructed using DNA recombinase (G-force Enzyme) (Vazyme, Nanjing, China) (Appendix A Table A7). The recombinant product was transformed into *E. coli* GT115 competent cells using the heat shock method, spread onto LB medium containing kanamycin, and incubated in an inverted position for 12–16 h. Once a single colony had grown, it was picked for colony PCR analysis. The primers used were U6p.4-F and gRNA-R, and the clone was tested by digestion with the *Xba*I restriction enzyme; clones that remained undigested were considered positive. Positive clones were selected and sent to Shanghai Geneworks for sequencing. The genomic regions encompassing both the sgRNA1 and sgRNA2 target sites were PCR-amplified and sequenced, and the resulting sequences were compared with the wild-type *LbMLO6* sequence to identify mutations at each target site.

A total of 500 seeds of Ningqi-1 were disinfected with 70% ethanol for 1 min, disinfected with 2.5% sodium hypochlorite for 30 s, and with 0.1% HgCl_2_ for 30 s. Following disinfection, the seeds were rinsed several times with sterile water, air-dried, and transferred with forceps to MS medium. They were then cultured in the plant tissue culture laboratory for approximately 20 days until the seedlings had developed 4–6 true leaves, at which point they were used for genetic transformation of *Lycium*. The GV3101 *Agrobacterium* glycerol stock (Biyuntian Biotechnology (Shanghai) Co., Ltd.) (Beyotime Biotechnology, Shanghai, China)stored at −80 °C was thawed on ice, streaked onto agar, and once single colonies had formed, a single colony was picked and inoculated into LB liquid medium for subculturing until the OD_600_ reached 0.6–0.8. A total of 50 mL of the bacterial suspension was taken, centrifuged at 6000 rpm for 10 min, the supernatant was discarded, and the bacterial cells were resuspended in 5 mL of pre-chilled CaCl_2_. This step was repeated once to prepare GV3101 competent *Agrobacterium*. The bacterial suspension was aliquoted and store at −80 °C. A volume of 2 μL of the constructed recombinant vector was taken and added to 50 μL of pre-thawed GV3101 *Agrobacterium* competent cells. Using the heat shock method, the recombinant plasmid was transformed into the competent cells. A total of 120 μL was spread onto LB medium (containing 50 mg/L kanamycin and 50 mg/L rifampicin), then the plate was incubated upside down for 36 h. A single colony was isolated for PCR analysis; successfully amplified colonies were subcultured to a volume of 100 mL. The bacterial suspension was centrifuged for 10 min to collect the cells, resuspended twice in sterile water to wash off the LB medium, then MS liquid medium was added and the concentration was adjusted to an OD_600_ of 0.8 and set aside. A 1 cm section was cut from the hypocotyl of a Ningqi-1 seedling, which was wounded to facilitate infection, and placed separately in pre-culture media for leaves and stem segments (2.74 g/L 1/2MS + 1.0 mg/L 6-BA + 0.2 mg/L NAA + 30 g/L sucrose + 6 g/L agar) for 48 h. Upon completion of pre-culture, they were transferred to the bacterial suspension for infection for 10 min. After infection, they were rinsed once with sterile water, the surface was blotted dry with sterile filter paper and placed on co-culture medium (2.74 g/L 1/2 MS + 1.0 mg/L 6-BA + 0.2 mg/L NAA + 30 g/L sucrose + 6 g/L agar). They were incubated in the dark at 25 ± 2 °C for 2 days and observed once every 6 h during this period; if a bacterial biofilm appeared, the hypocotyl or leaf was repositioned. Upon completion of the co-culture, the samples were washed and sterilized with sterile water and antibiotics, then placed on a screening medium ((2.74 g/L ½ MS + 1.0 mg/mL 6-BA + 0.2 mg/mL NAA + 30 g/L sucrose + 6 g/L agar + 200 mg/L cefuroxime + 30 mg/L nystatin). The medium was replaced every 20 days until adventitious buds emerged, at which point the plants were transferred to hormone-free medium for root formation.

Approximately 0.5 g of rooted seedlings was taken from the *Lbmlo6* mutant line and the DNA was extracted from the plants using a genomic DNA extraction kit (Tiangen Biotech, Beijing, China). PCR amplification was performed using the extracted DNA as the template, primers Xncas9-F and Xncas9-R, and Taq PCR mix to determine whether the vector had been introduced into the *Lbmlo6* mutant lines. The samples were then sent to Shanghai Sangon Biotech for sequencing; the *Lbmlo6* sequence was compared with the sequencing results to determine the type of deletion. Representative *Lbmlo6* edited lines and regenerated non-edited Ningqi-1 plants were inoculated with the powdery mildew pathogen under the same conditions. The non-edited CK plants underwent the same tissue-culture and regeneration procedures as the edited plants but showed no detectable editing at the *LbMLO6* target sites. Powdery mildew infection was qualitatively evaluated by trypan blue staining at 12, 24, and 48 h post-inoculation.

## 5. Conclusions

Powdery mildew infection induced extensive molecular reprogramming in *Lycium barbarum*. Across the three post-inoculation comparisons, 1299 shared DEGs, 75 shared DEPs, and 127 shared DAMs were identified. Integrated multi-omics analysis indicated coordinated changes in plant–pathogen interaction, hormone-associated signaling, phenylpropanoid metabolism, α-linolenic acid metabolism, and other defense-related processes. The non-parallel changes observed at the transcript, protein, and metabolite levels further suggest that the response to powdery mildew involves multi-layered regulation rather than a simple one-to-one correspondence among the three omics levels.

In addition, functional analysis of *LbMLO6* using CRISPR/Cas9-mediated gene editing provided preliminary evidence for its involvement in powdery mildew susceptibility. Mutations were detected in 21 of 24 candidate regenerated lines, corresponding to an editing efficiency of 87.5%. Qualitative microscopic observations revealed differences in fungal development between representative *Lbmlo6* edited lines and the control following inoculation. These findings provide preliminary evidence that *LbMLO6* may be associated with the powdery mildew response; however, quantitative assessment of fungal biomass and disease severity is required to determine whether its disruption reduces powdery mildew susceptibility.

However, quantitative assessment of fungal biomass and disease severity, inclusion of empty-vector transformed controls, analysis of genetically stable independent edited lines, off-target evaluation, genetic stability assessment, functional complementation, and field-level validation will be required to establish the specific contribution of *LbMLO6* to powdery mildew resistance.

## Figures and Tables

**Table 1:** Mutation patterns identified in CRISPR/Cas9-edited *Lbmlo6* lines.

Mutation Type	Mutant Plant	Proportion of Edited Lines (%)	Whether Homozygous Mutation
A/T insertion	2, 3, 4, 5, 6, 7, 8, 9, 10, 11, 12, 13, 19, 20, 21	71.4	Yes
T deletion	15, 17, 24	14.2	No
AC insertion	16	4.76	No
Large deletion	1, 22	9.52	No

**Table 2:** Leaf area-related parameters of plants from the *Lbmlo6* mutant line.

Number	Length of Leaf (cm)	Maximum Width (cm)	Blade Thickness (mm)	Leaf Area (cm^2^)	Length-Width Ratio
CK	4.2926 ± 0.44 a	2.2 ± 0.15 a	0.24 ± 0.214 ab	14.79 ± 4.49 a	1.99 ± 0.32 a
5	5.0694 ± 0.41 a	1.4 ± 0.15 bc	0.2 ± 0 b	7.18 ± 1.08 ab	4.01 ± 0.32 ab
10	4.9547 ± 0.41 a	1.76 ± 0.1 ab	0.2 ± 0 b	12.66 ± 1.60 ab	2.84 ± 0.430 ab
11	4.4187 ± 0.45 a	1.56 ± 0.06 b	0.3 ± 0 a	15.24 ± 3.99 a	2.80 ± 0.19 a
2	3.7032 ± 0.22 a	0.96 ± 0.088 c	0.13 ± 0.033 c	3.43 ± 0.3 b	3.86 ± 0.214 b

Note: Values are presented as mean ± SE (*n* = 5). Different lowercase letters within the same column indicate significant differences among groups according to one-way ANOVA followed by Tukey’s HSD test (*p* < 0.05). Leaf length and maximum width are expressed in cm, blade thickness in mm, leaf area in cm^2^, and the length-to-width ratio is dimensionless.

## Data Availability

The data presented in this study are available upon request from the corresponding author.

## Supplementary Materials

The following supporting information can be downloaded at: https://www.mdpi.com/article/10.3390/ijms27219412/s1.

## Appendix A

**Table A1:** qPCR primer sequences.

GeneID	Name	Sequence (5′-3′)	Sequence (3′-5′)
*Lru01G017344.1*	*CML35*	F:CAATTGGGAATGGGAAGGCT	R:ACCCCATGTTGTAGTCCTGA
*Lru01G048735.1*	*WRKY70*	F:CCCGTATGTGCAAGGATGAA	R:TCTCCATCAACTCCACCCAT
*Lru01G011568.1*	*CATA12*	F:ACCCCTCAATCACCAAAACC	R:GGTTCAGGATCGCATTGGAT
*Lru01G049867.1*	*F-box*	F:GCTTAGCTATGGTGGGGAAC	R:CGAGACCTTGGAATTGCTGA
*Lru01G019510.1*	*CAMT3*	F:TTGCCATGGATATCAACCGG	R:TTGTGGATGCTGACAAGGAC
*Lru01G040980.1*	*GLOSSY1*	F:CTCGAAGCCTCATGACAACA	R:TGGAAGCAGATCAGAAAGGC
*Lru01G007387.1*	*CYP450*	F:TACCCGTCCGAGAGAAAACT	R:AACGATGTCATTTGGGTGCT
*Lru01G026673.1*	*NPR*	F:GCAACAAAAGCGACGGTTAA	R:TGAGAAGAATGCCACAGTGG
*Lru01G039592.1*	*MIOX*	F:ACGATGTACCCTTGGGAAGA	R:CCATGGACAAAACATTCGCC
*Lru01G041208.1*	*Chts*	F:ACCCCTCAATCACCAAAACC	R:GGTTCAGGATCGCATTGGAT
*Lru01G039198.1*	*CAD1*	F:GGTGGCTTGGAAGTTGAGAA	R:TGAAGTAGTGGAGGTAGGCC
*novel.5673*	*CML49*	F:CTGAGTTTTGGGATGGTGCT	R:GGCTACAAACAGGGTTCCAA
Reference gene	*Actin1*	F:GGAAACATAGTGCTCAGTGGTG	R:GCTGAGGGAAGCCAAGATAG
	*GAPDH*	F:GACAACTACTCACTCTTACACCG	R:GTTCGGGAGGACAAGGGAAA

**Table A2:** UPLC–MS/MS sampling quantities and analytical conditions.

Proportion		Time	Condition
Phase A (ultrapure water (with 0.1 per cent formic acid added))	Phase B (acetonitrile (with 0.1 per cent formic acid))		
95%	5%	0 min	Flow rate: 0.35 mL/min; Column temperature: 40 °C; Injection volume: 4 μL
95–5%	5–95%	9 min	
5%	95%	1 min	
5–95%	95–5%	1 min	
95%	5%	3 min	

**Table A3:** Summary of MRM acquisition, ionization, relative quantification, and metabolite-identification parameters documented in the metabolomics report.

Parameter/Item	Information	Identification Criterion
Metabolite annotation	Metabolites were qualitatively and quantitatively analyzed based on an in-house/local metabolite database	Database-assisted metabolite annotation
Detection mode	Multiple reaction monitoring (MRM) was used for metabolite detection	MRM-based metabolite detection and relative quantification
Ion selection	A triple-quadrupole system was used to select characteristic ions for each metabolite, and the detector recorded the characteristic-ion signal intensity (CPS)	Metabolite-specific characteristic-ion selection by triple-quadrupole mass spectrometry
Q1 (Da)	Defined in the report as the precursor/parent ion after electrospray ionization	Metabolite-specific precursor (parent) ion
Q3 (Da)	Defined in the report as the characteristic fragment ion	Metabolite-specific characteristic fragment (product) ion
Ionization model	The report defines M + H as positively charged and M-H as negatively charged	Positive- or negative-ionization mode assignment ([M + H]^+^ or [M − H]^−^)
Relative quantification	MultiQuant was used for chromatographic peak integration and correction. The integrated peak area (Area) represented the relative abundance of the corresponding metabolite	Relative abundance based on integrated chromatographic peak area
Identification level 1	Sample MS/MS spectrum (all fragment ions) and RT matched the database with a score > 0.7	MS/MS- and RT-based database match with a score > 0.7
Identification level 2	Sample MS/MS spectrum (all fragment ions) and RT matched the database with a score of 0.5–0.7	MS/MS- and RT-based database match with a score of 0.5–0.7
Identification level 3	Sample Q1, Q3, RT, DP, and CE were consistent with the database	Database match based on consistency of Q1, Q3, RT, DP, and CE

**Table A4:** PCR amplification procedure.

Temperature (°C)	Time	Cycle
94	5 min	1×
94	30 s	30×
58	30 s	
68	45 s	
68	10 min	1×

**Table A5:** Primers for gene knockout vector construction.

Primer Name	Primer Sequences	Number of Bases (bp)	Effect
*MLO6-F*	ATGGCTGGTGGTGGGGGT	18	*MLO* cloning upstream primer
*MLO6-R*	CTATTGTAAACCGAAATTCAATGC	24	*MLO* cloning downstream primer
*U6p.4-F*	CAGGAAACAGCTATGACCATATTCATTCGGAGTTTTTGTATC	42	Universal primers upstream of SgRNA
*gRNA-R*	AGCACCGACTCGGTGCCAC	19	Universal primers downstream of SgRNA
*SgRNA1-R*	GAGCATATTATCCACCTCAT	20	Primer 1 downstream of the target sequence
*SgRNA2-R*	GTACCATTTGTGTCT	15	Primer 2 downstream of the target sequence
*Hygjc2-F*	GTCCGTCAGGACATTGTTGGAGCC	24	Upstream primer for the detection of hygromycin resistance genes
*Hygjc2-R*	GTCTCCGACCTGATGCAGCTCTCGG	25	Downstream primers for the detection of hygromycin resistance genes
*xncas9-F*	CCTGTTCGGGAATCTCATTGC	21	Upstream primers for Cas9 gene detection
*xncas9-R*	TGAATCTGGTGTGGGATGCTG	21	Downstream primers for Cas9 gene detection

**Table A6:** Bioinformatics analysis software.

Tool	Website	Function
ProParam	https://web.expasy.org/protparam/ (accessed on 18 October 2026)	Analysis of the Physicochemical Properties of Proteins
Cell-PLoc-2/	http://www.csbio.sjtu.edu.cn/bioinf/Cell-PLoc-2/ (accessed on 18 October 2026)	Subcellular localization
TMHMM2.0 server	http://services.healthtech.dtu.dk/service.php?TMHMM-2.0 (accessed on 18 October 2026)	Prediction of transmembrane domains
SignalP 5.0	http://www.cbs.dtu.dk/services/singnalP/ (accessed on 18 October 2026)	Signal Peptide Analysis
NPS@	https://npsa-prabi.ibcp.fr/cgi-bin/npsa_automat.pl?page=/NPSA/npsa_hnn.html (accessed on 18 October 2026)	Second-level structure prediction
Uniprot	https://www.uniprot.org/	Three-level structure prediction
MEME	https://meme-suite.org	Structure-Function Domain Analysis

**Table A7:** Components and amounts used for gene-editing vector construction.

Component	Sample
DNA recombinase	1 μL
Linearized pP1C.4 vector	3–5 μL
SgRNA	3–4 μL
ddH_2_O	Add to 10 μL

**Table A8:** Statistical analysis of transcriptome sequencing data.

Simple	Raw Reads	Clean Reads	Clean Base (G)	Q20 (%)	Q30 (%)	GC Content (%)
CK-1	46865722	45459352	6.82	97.42	92.74	42.79
CK-2	49530404	48153170	7.22	97.5	92.92	42.8
CK-3	43695000	42275978	6.34	97.28	92.45	42.84
12 h-1	46314868	44701054	6.71	97.43	92.78	43.06
12 h-2	49118422	47877662	7.18	97.42	92.67	43.2
12 h-3	43434578	41916672	6.29	97.3	92.51	43.09
24 h-1	49032366	47323798	7.1	97.45	92.8	42.57
24 h-2	48595028	46525706	6.98	97.49	92.84	42.25
24 h-3	47659748	45632660	6.84	97.26	92.39	42.45
48 h-1	48505224	46858126	7.03	97.53	93.02	43.43
48 h-2	44303808	43020580	6.45	97.34	92.58	43.38
48 h-3	44103172	42622410	6.39	97.19	92.25	43.15

**Table A9:** Alignment statistics of transcriptome samples against the *Lycium barbarum* reference genome.

Sample	Total Reads	Reads Mapped	Unique Mapped	Read1 Mapped	Read2 Mapped
CK-1	45459352	37012429	33742492	16849967	16892525
		(81.42%)	(74.23%)	(37.07%)	(37.16%)
CK-2	48153170	39251221	35758269	17850925	17907344
		(81.51%)	(74.26%)	(37.07%)	(37.19%)
CK-3	42275978	33977636	30983969	15455721	15528248
		(80.37%)	(73.29%)	(36.56%)	(36.73%)
12 h-1	44701054	35912172	32599318	16241946	16357372
		(80.34%)	(72.93%)	(36.33%)	(36.59%)
12 h-2	47877662	38898088	35368842	17638013	17730829
		(81.24%)	(73.87%)	(36.84%)	(37.03%)
12 h-3	41916672	33862873	30703849	15302855	15400994
		(80.79%)	(73.25%)	(36.51%)	(36.74%)
24 h-1	47323798	36840683	33739552	16829453	16910099
		(77.85%)	(71.30%)	(35.56%)	(35.73%)
24 h-2	46525706	36222137	33175833	16543408	16632425
		(77.85%)	(71.31%)	(35.56%)	(35.75%)
24 h-3	45632660	35436121	32460153	16185806	16274347
		(77.66%)	(71.13%)	(35.47%)	(35.66%)
48 h-1	46858126	34182923	31348016	15628669	15719347
		(72.95%)	(66.90%)	(33.35%)	(33.55%)
48 h-2	43020580	31365114	28773372	14340497	14432875
		(72.91%)	(66.88%)	(33.33%)	(33.55%)
48 h-3	42622410	31883674	29278254	14586013	14692241
		(74.80%)	(68.69%)	(34.22%)	(34.47%)

Note: Sample: sample name; Total Reads: total number of clean reads; Reads Mapped: The number of reads mapped to the reference genome; Unique Mapped: The number of reads that are uniquely mapped against the reference genome; Read1 Mapped: number of read1 mapped; Read2 Mapped: number of read2 mapped.

**Table A10:** Significantly enriched KEGG pathways.

KEGG_Pathway	KEGG Number	Enriched Gene Number		
		12 h	24 h	48 h
Metabolic pathways	ko01100	503	825	918
Plant-pathogen interaction	ko04626	178	196	217
Plant hormone signal transduction	ko04075	129	172	202
MAPK signaling pathway-plant	ko04016	105	131	161
Phenylpropanoid biosynthesis	ko00940	70	113	114
Circadian rhythm-plant	ko04712	36	77	82
Tryptophan metabolism	ko00380	37	46	48
Amino sugar and nucleotide sugar metabolism	ko00520	26	35	45
Cysteine and methionine metabolism	ko00270	27	41	43
Zeatin biosynthesis	ko00908	26	31	39
Glyoxylate and dicarboxylate metabolism	ko00630	18	36	38
Flavonoid biosynthesis	ko00941	26	40	35
Cutin, suberine and wax biosynthesis	ko00073	30	35	33
Pyruvate metabolism	ko00620	25	33	33
Glycerolipid metabolism	ko00561	14	25	33
Biosynthesis of various plant secondary metabolites	ko00999	19	28	32
Carotenoid biosynthesis	ko00906	19	24	29
Other glycan degradation	ko00511	19	22	27
Stilbenoid, diarylheptanoid and gingerol biosynthesis	ko00945	11	24	23
Arginine and proline metabolism	ko00330	12	19	23
ABC transporters	ko02010	21	20	22
Ubiquinone and other terpenoid-quinone biosynthesis	ko00130	9	18	20
Fatty acid degradation	ko00071	12	17	18
Sesquiterpenoid and triterpenoid biosynthesis	ko00909	11	9	17
Pentose phosphate pathway	ko00030	9	15	17
Valine, leucine and isoleucine degradation	ko00280	11	16	17
Glucosinolate biosynthesis	ko00966	12	14	14
Tropane, piperidine and pyridine alkaloid biosynthesis	ko00960	12	14	14
Tyrosine metabolism	ko00350	8	15	13
Butanoate metabolism	ko00650	11	12	12
Diterpenoid biosynthesis	ko00904	7	16	10
Anthocyanin biosynthesis	ko00942	8	7	9
Selenocompound metabolism	ko00450	5	7	7
Monoterpenoid biosynthesis	ko00902	5	5	6
Indole alkaloid biosynthesis	ko00901	6	7	5
Glycosphingolipid biosynthesis-globo	ko00603	2	4	3
Limonene and pinene degradation	ko00903	2	2	2
Glycosphingolipid biosynthesis-lacto and neolacto	ko00601	1	2	1
