# Supplementary material for: Integrated Multi-Omics Analysis of the Response of Lycium barbarum ‘Ningqi-1’ to Powdery Mildew Infection and Functional Characterization of LbMLO6
Source: Int J Mol Sci. 2026 Oct 22;27(21):9412. doi: 10.3390/ijms27219412 (PMC13762972; doi:10.3390/ijms27219412)
Supplement: Supplementary file 1 [file ijms-27-09412-s001.zip › ijms-4622805-supplementary.pdf]

(a)

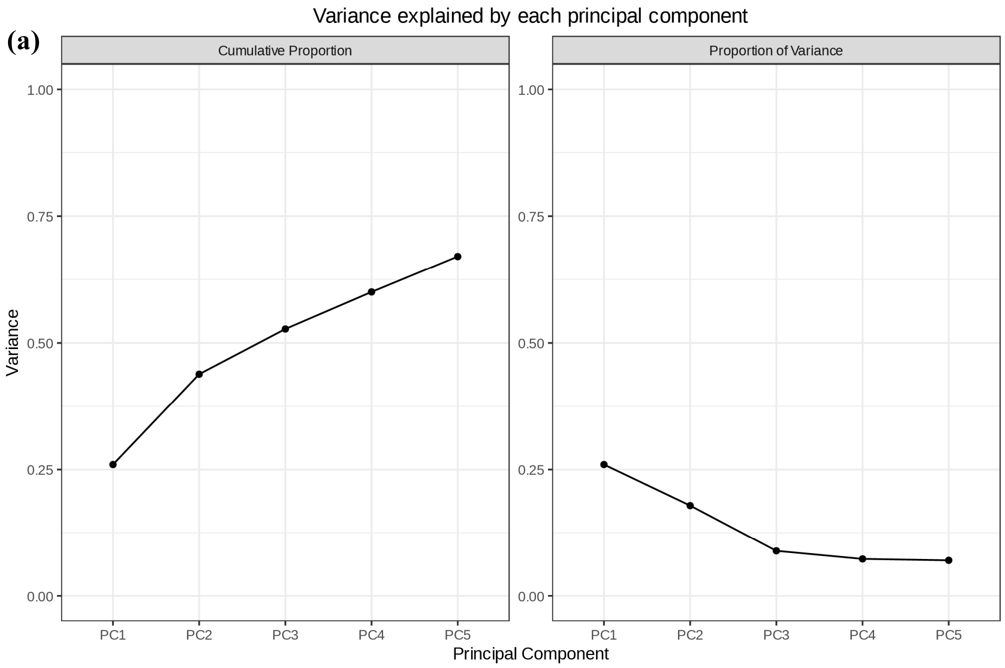

(b)

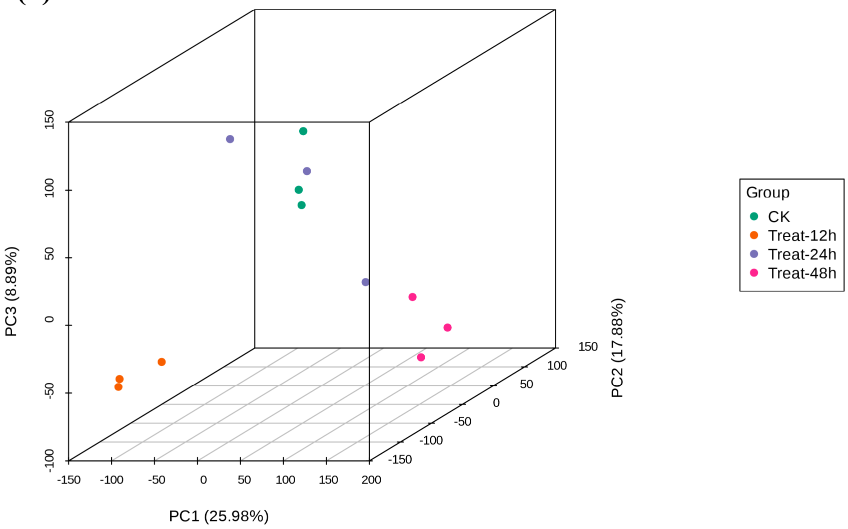

Supplementary Figure S1. Principal component analysis (PCA) of the transcriptomic data. (a) Scree plot showing the proportion of variance explained by each principal component; (b) Three-dimensional PCA score plot of all samples based on the first three principal components.

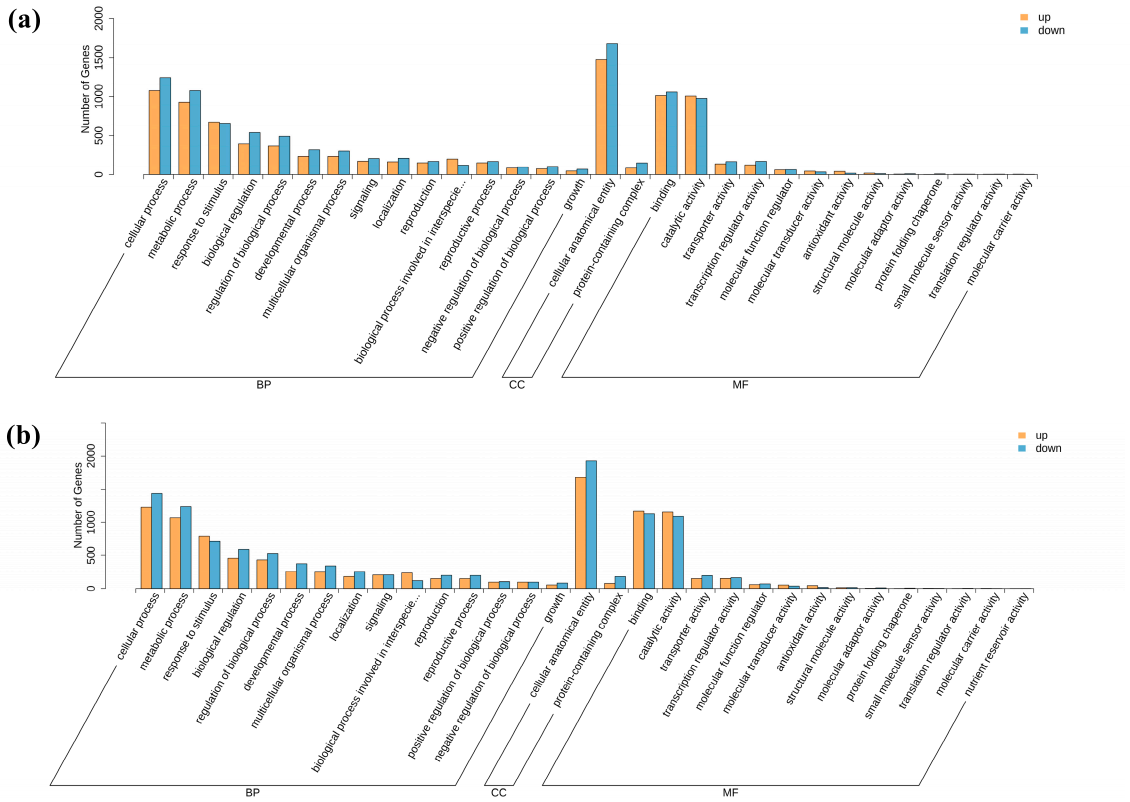

Supplementary Figure S2. Transcriptome GO enrichment analysis. (a) is CK vs 24 h; (b) indicates CK vs 48 h.

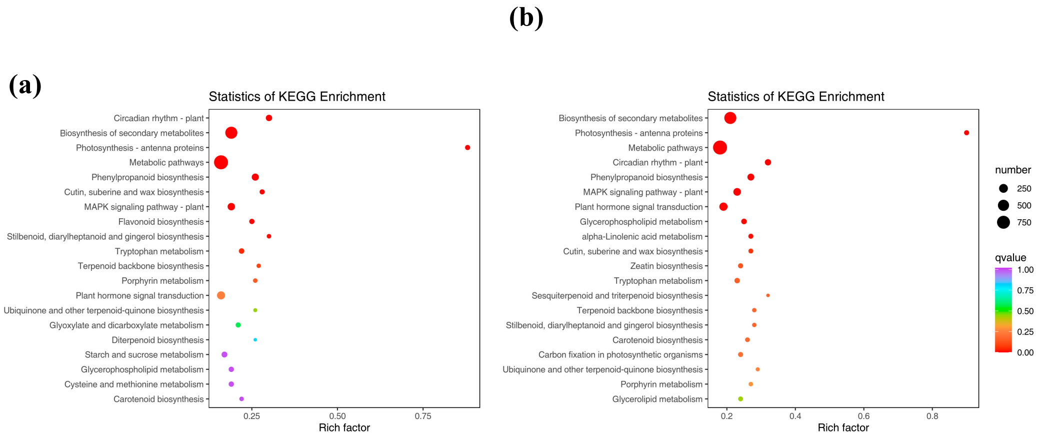

Supplementary Figure S3. KEGG enrichment analysis of the transcriptome. (a) is CK vs 24 h; (b) indicates CK vs 48 h.

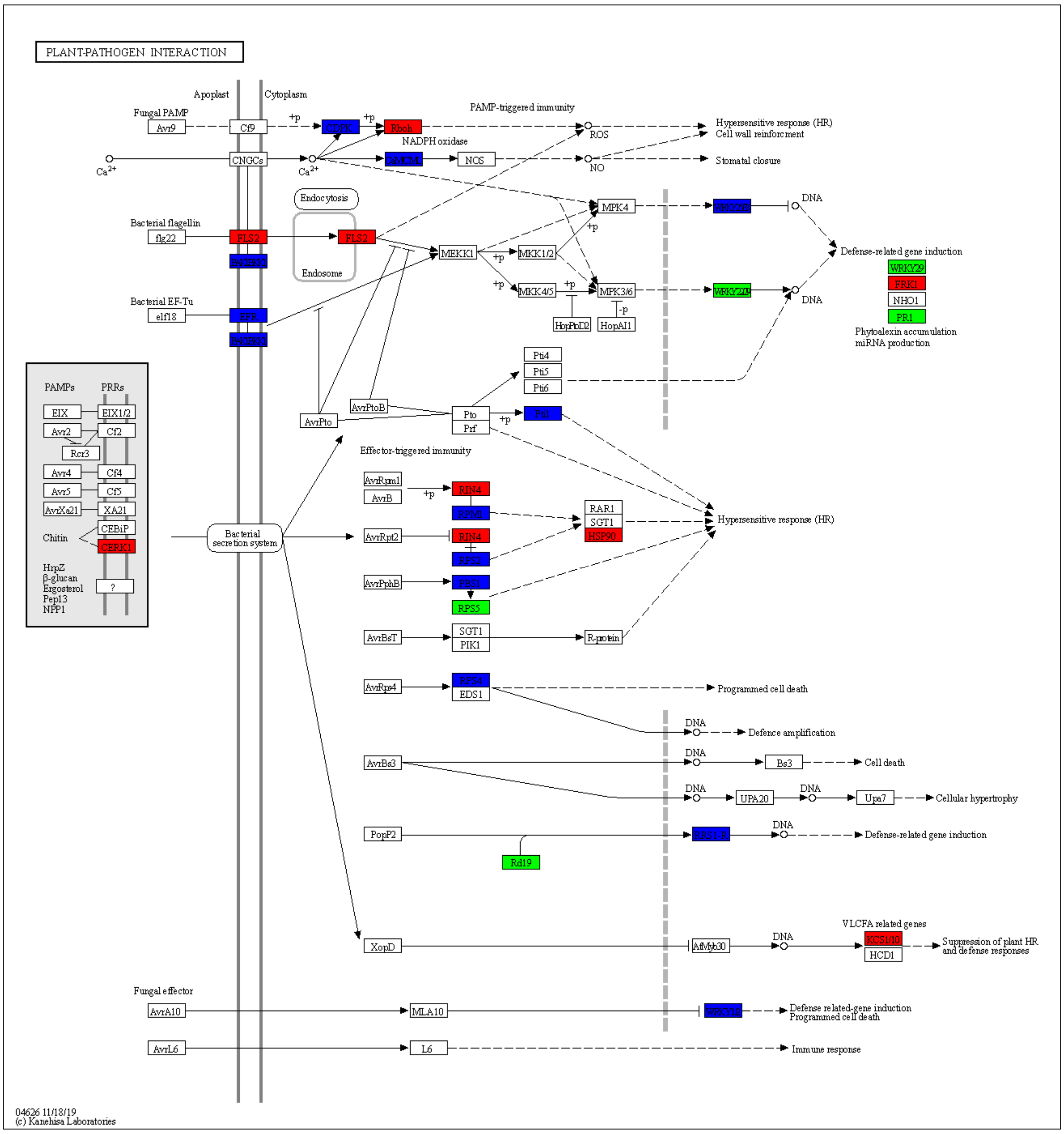

Supplementary Figure S4. Plant – pathogen interaction pathway.

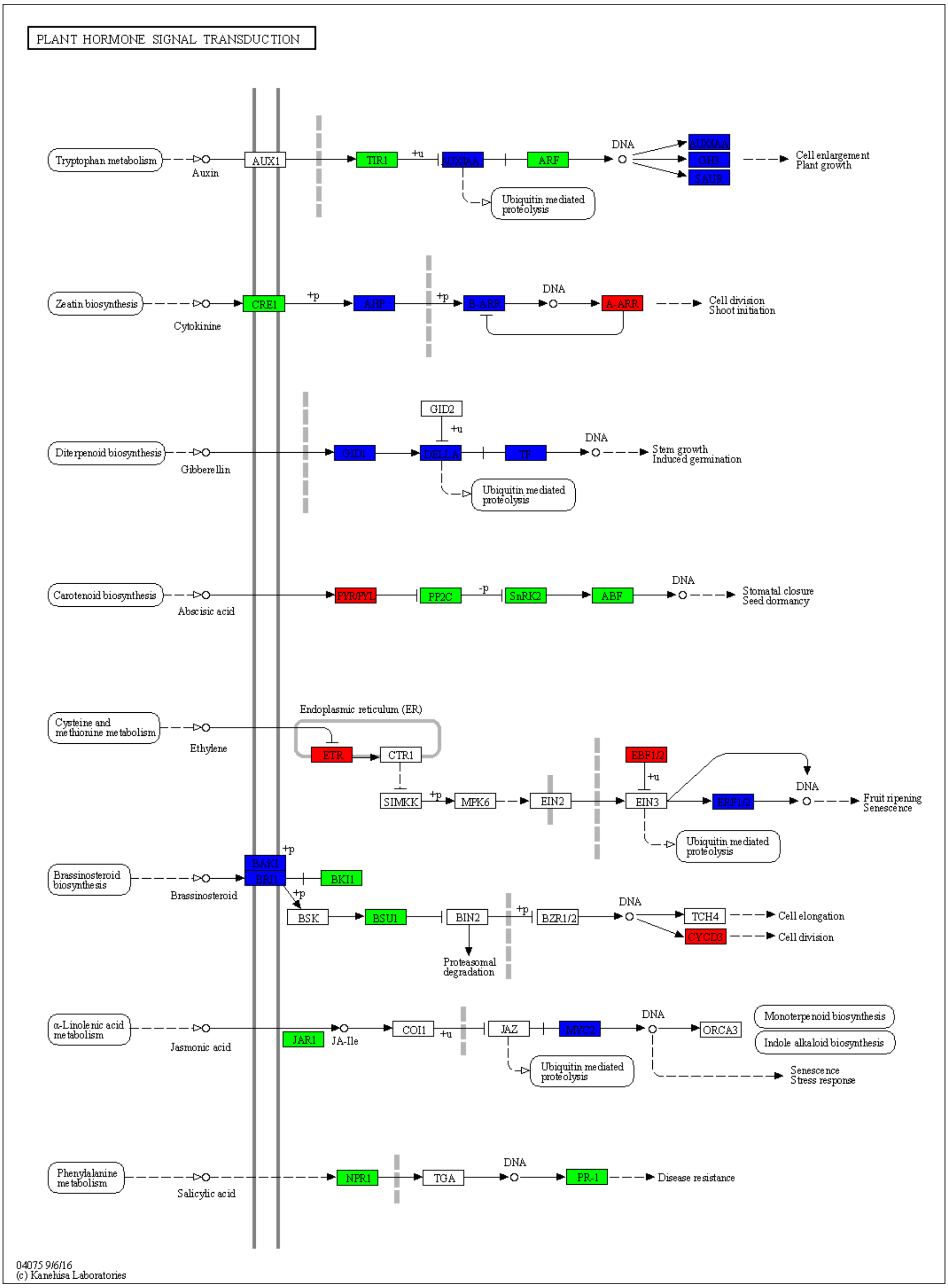

Supplementary Figure S5. Plant hormone signal transduction pathway.

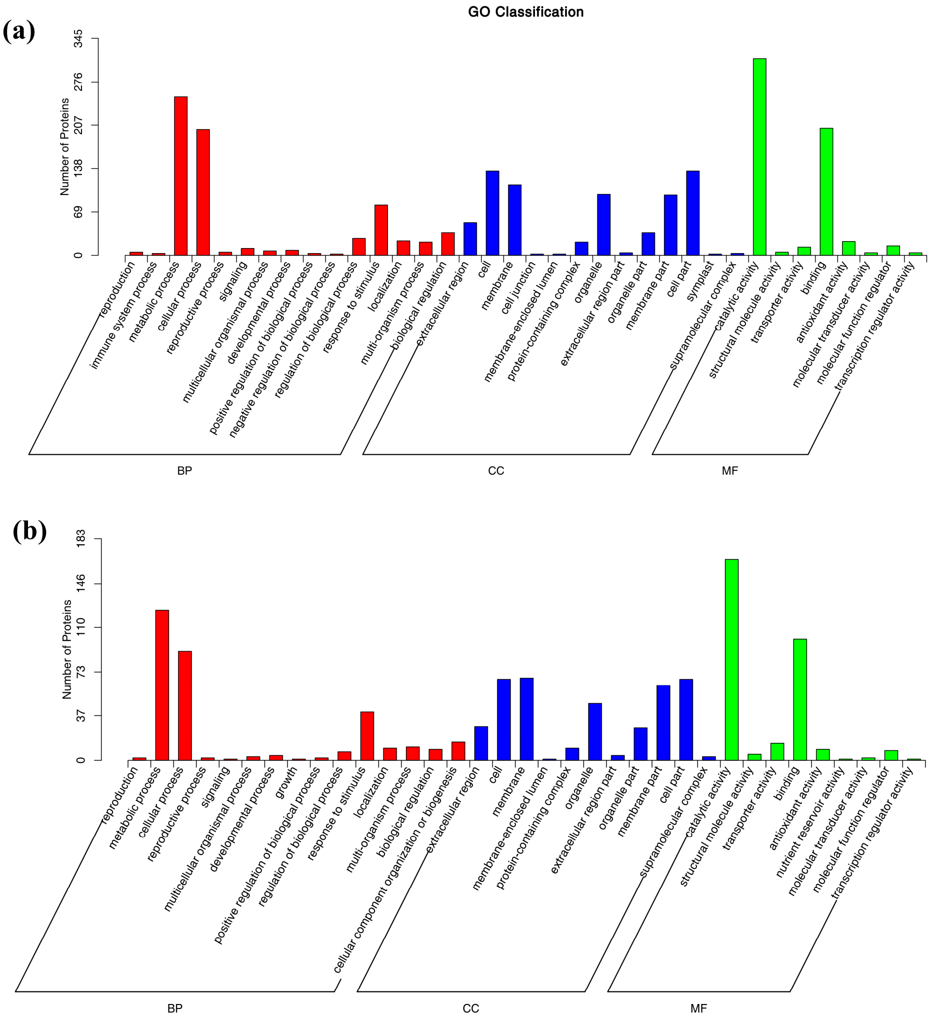

Supplementary Figure S6. Proteome GO enrichment analysi. (a) is CK vs 24 h; (b) indicates CK vs 48 h.

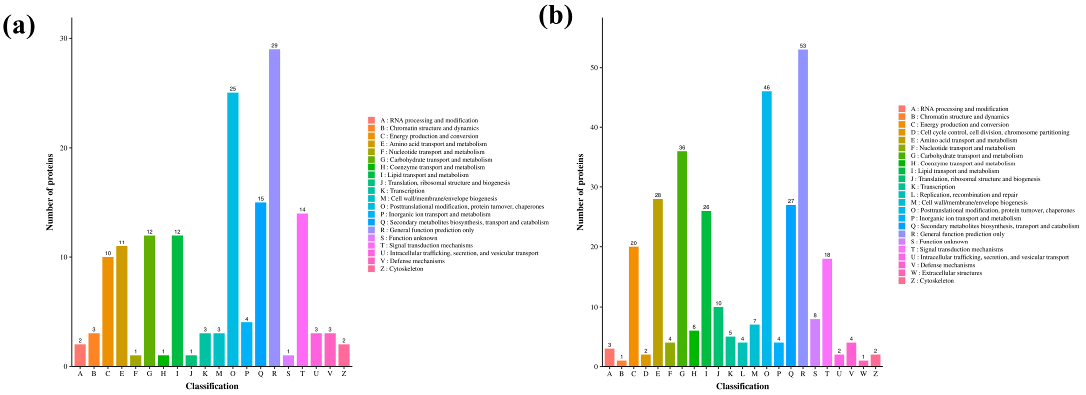

Supplementary Figure S7. KOG enrichment analysis of the proteome. (a) is CK vs 24 h; (b) indicates CK vs 48 h.

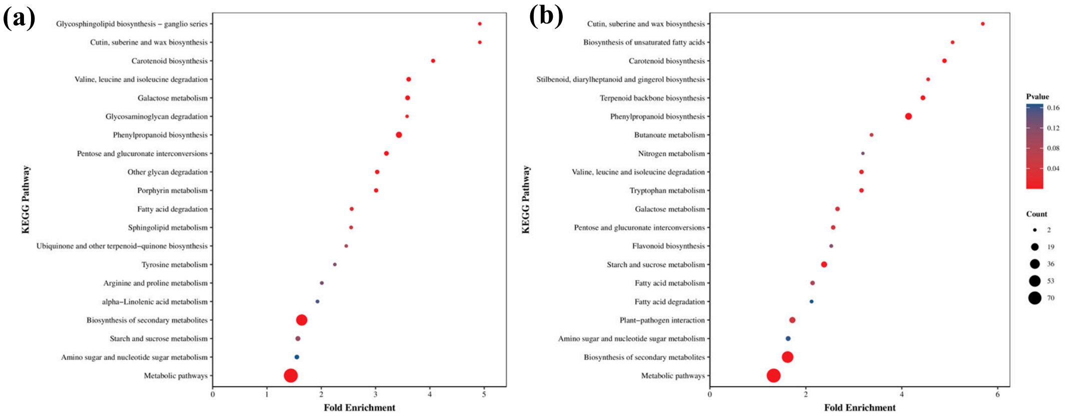

Supplementary Figure S8. KEGG enrichment analysis of the proteome. (a) is CK vs 24 h; (b) indicates CK vs 48 h.

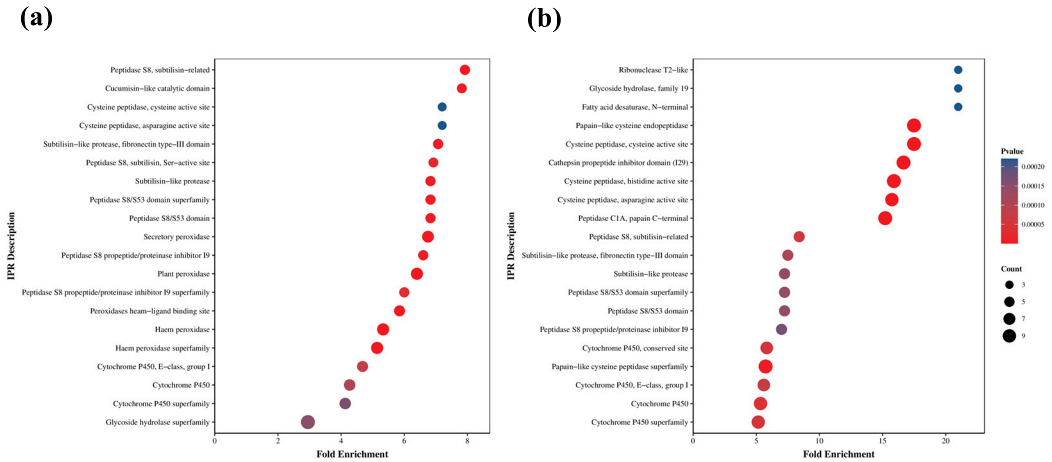

Supplementary Figure S9. IPR enrichment analysis of the proteome. (a) is CK vs 24 h; (b) indicates CK vs 48 h.

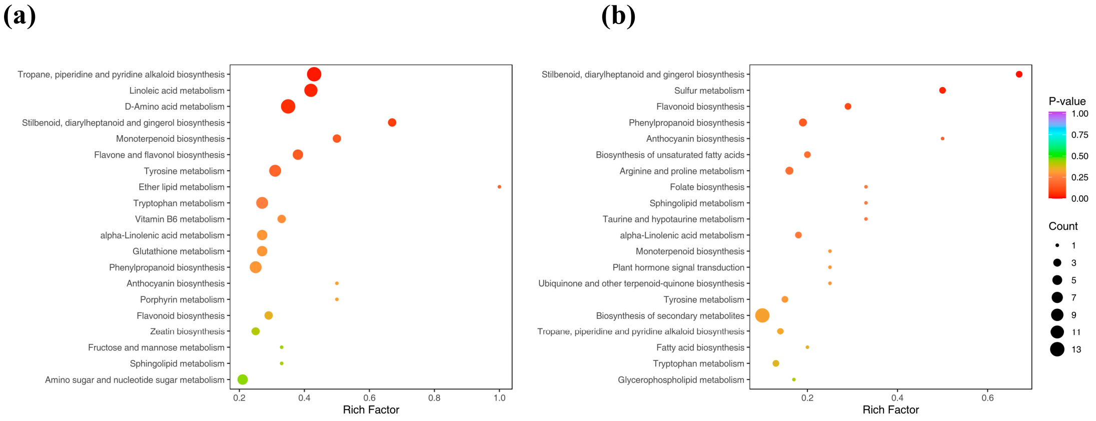

Supplementary Figure S10. KEGG enrichment analysis of the metabolome. (a) is CK vs 24 h; (b) indicates CK vs 48 h.

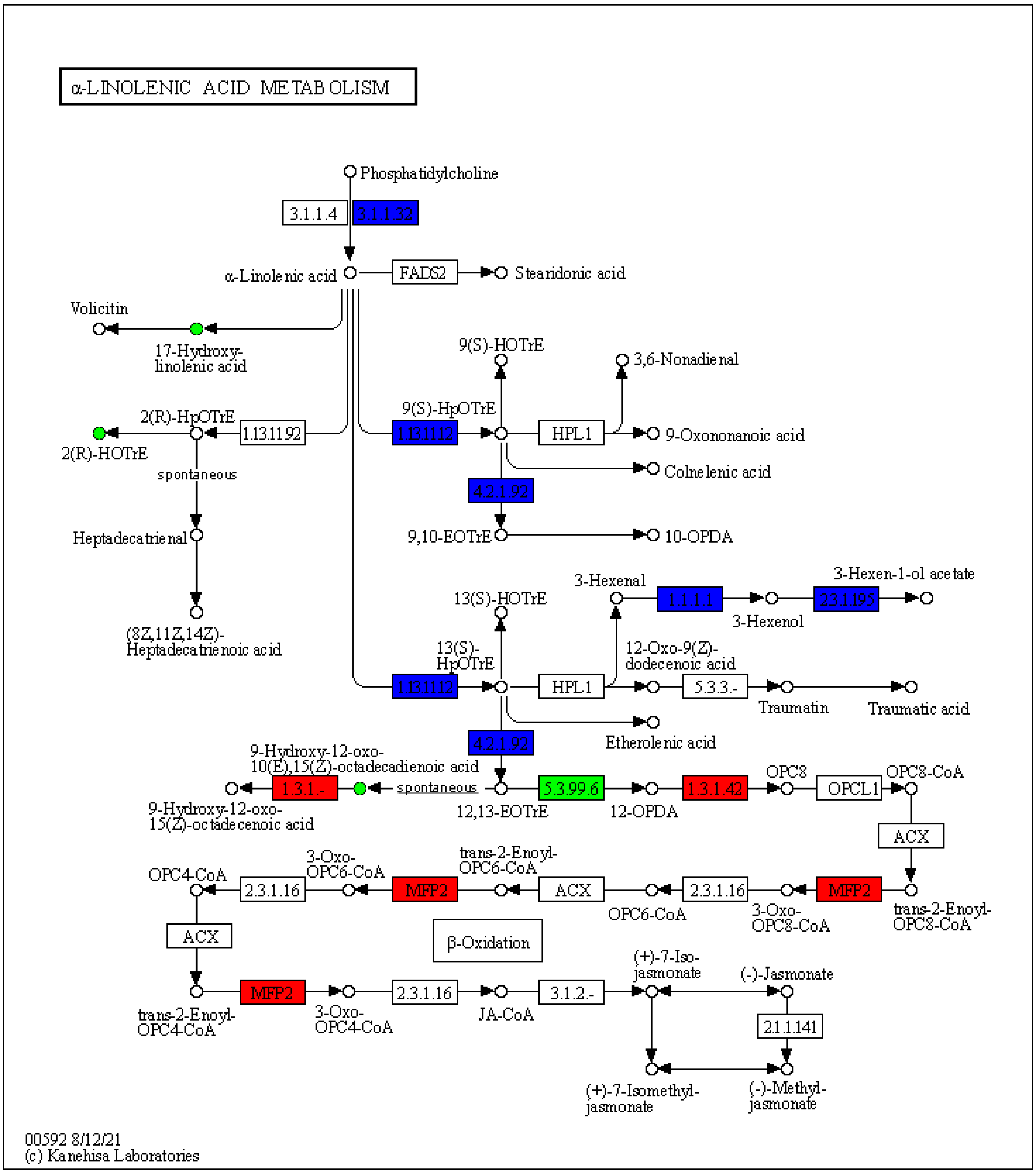

Supplementary Figure S11. α -Linolenic acid metabolism pathway.

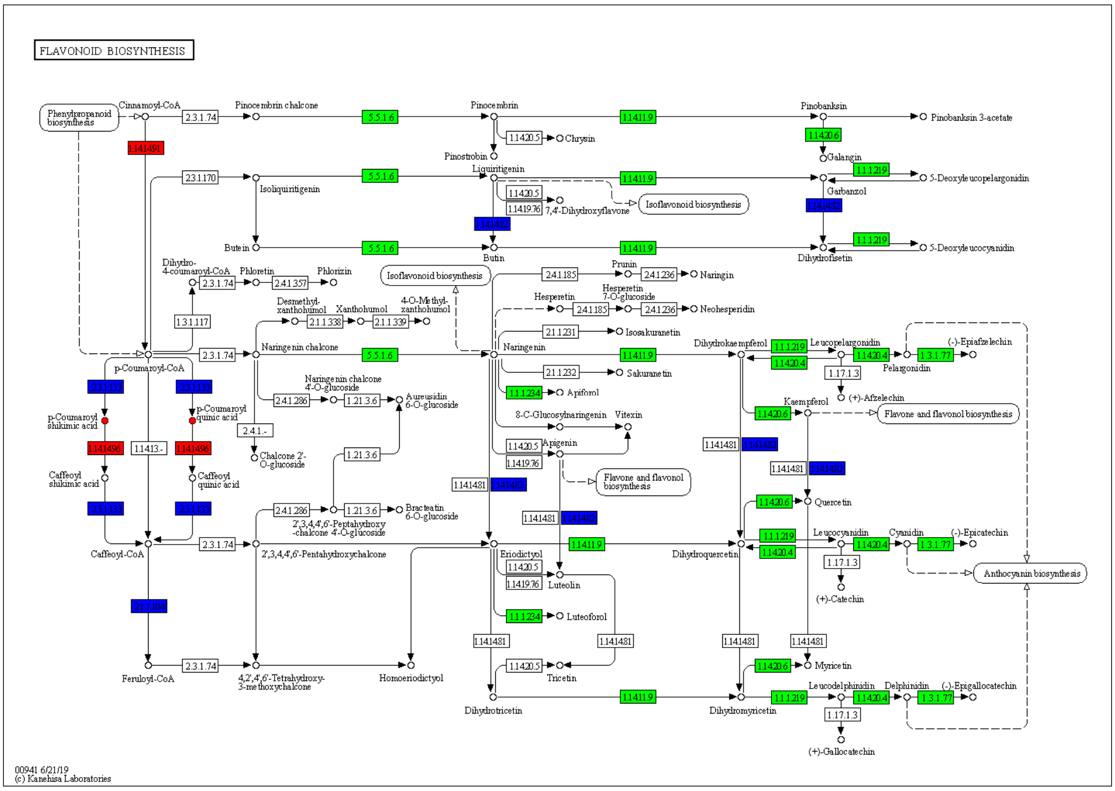

Supplementary Figure S12. Phenylpropanoid biosynthesis pathway.

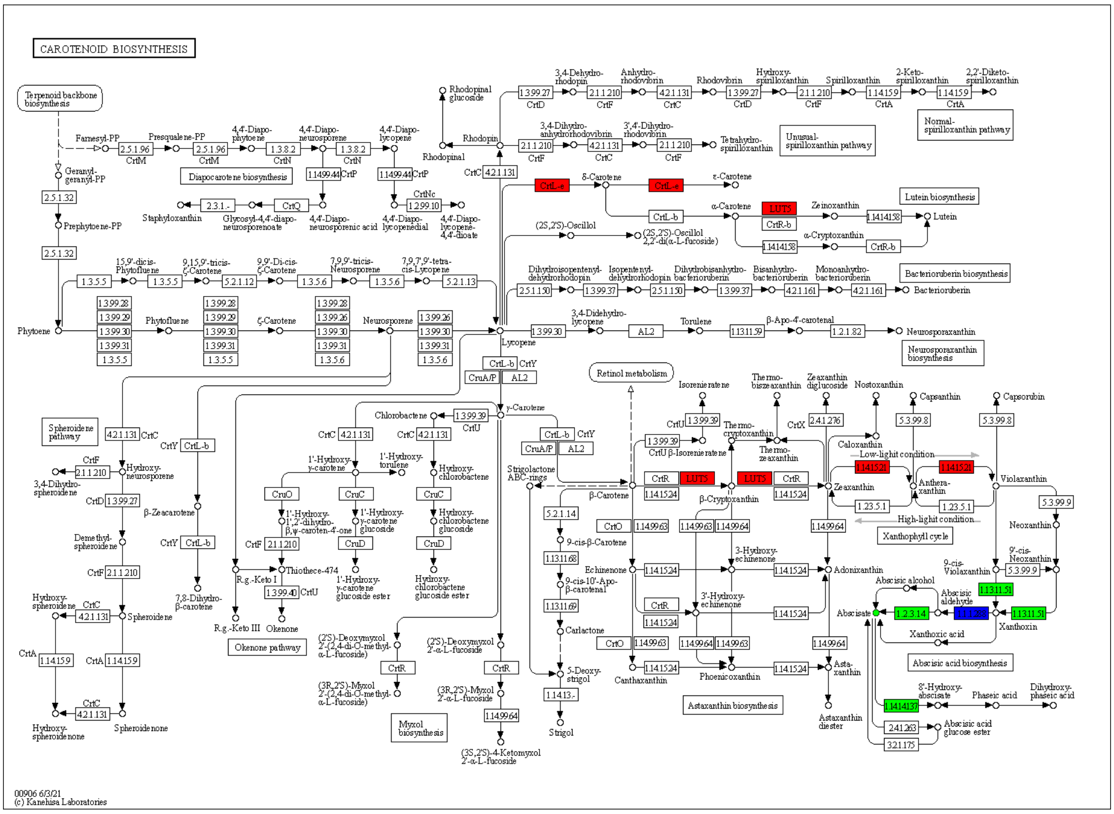

Supplementary Figure S13. Carotenoid biosynthesis pathway.

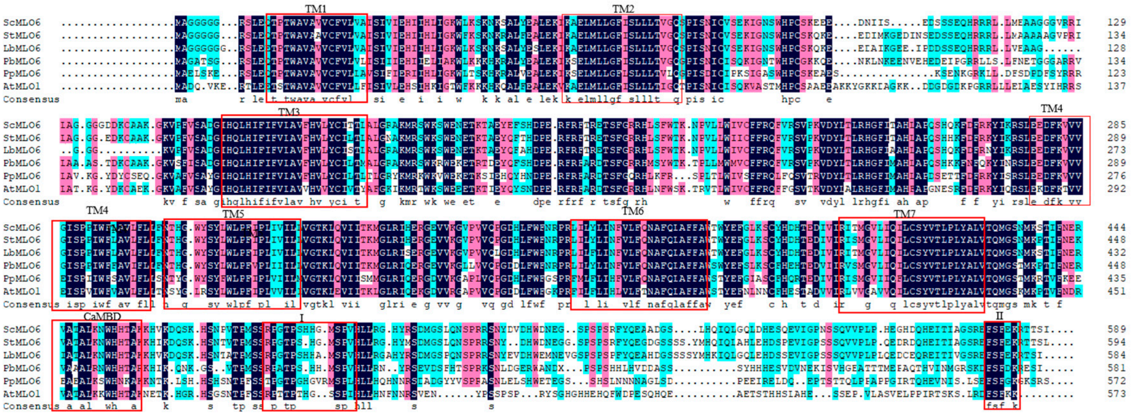

Supplementary Figure S14. Multiple sequence alignment of LbMLO6 with MLO6 proteins from other plant species.

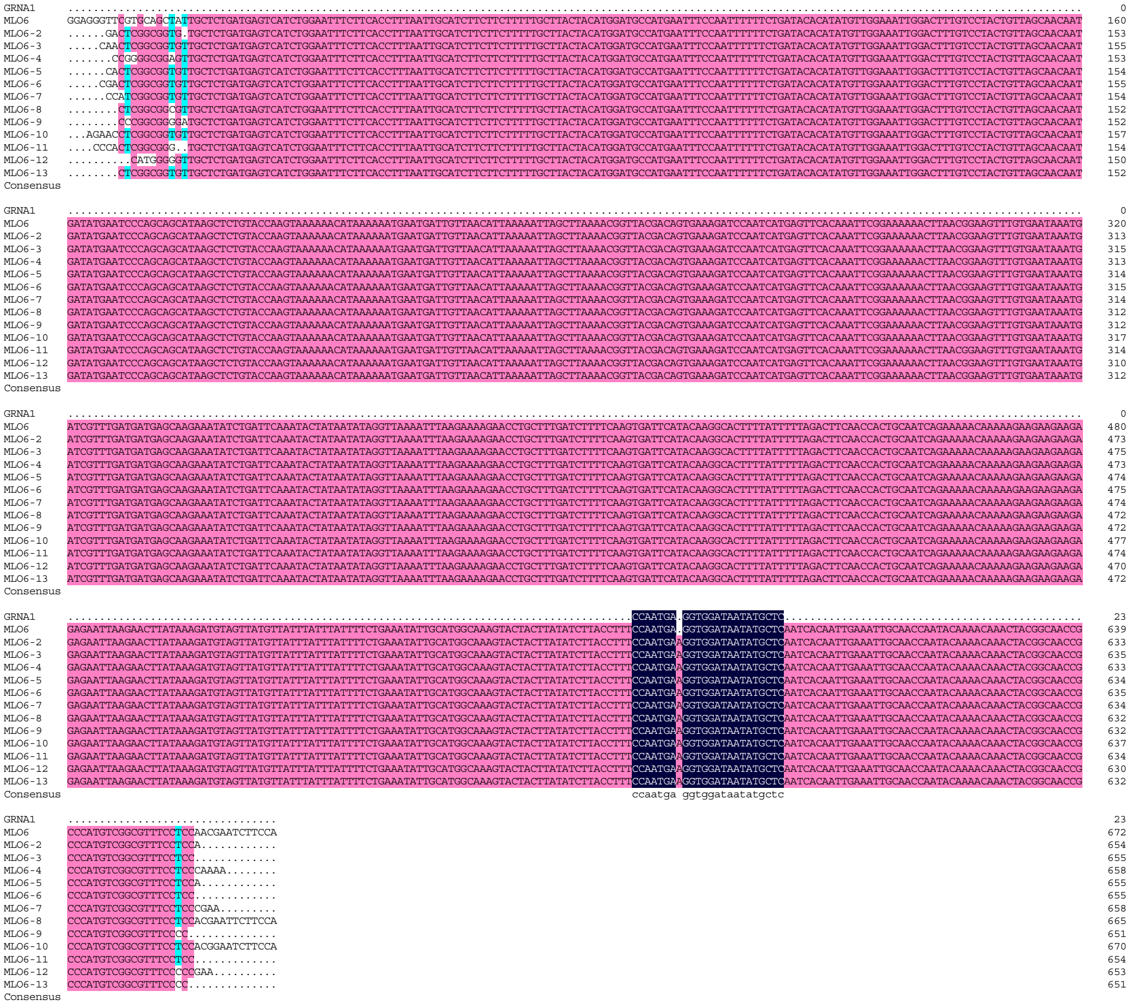

Supplementary Figure S15. Sequencing results of the CRISPR/Cas9-mediated *Lbmlo6* mutant line 2-13.

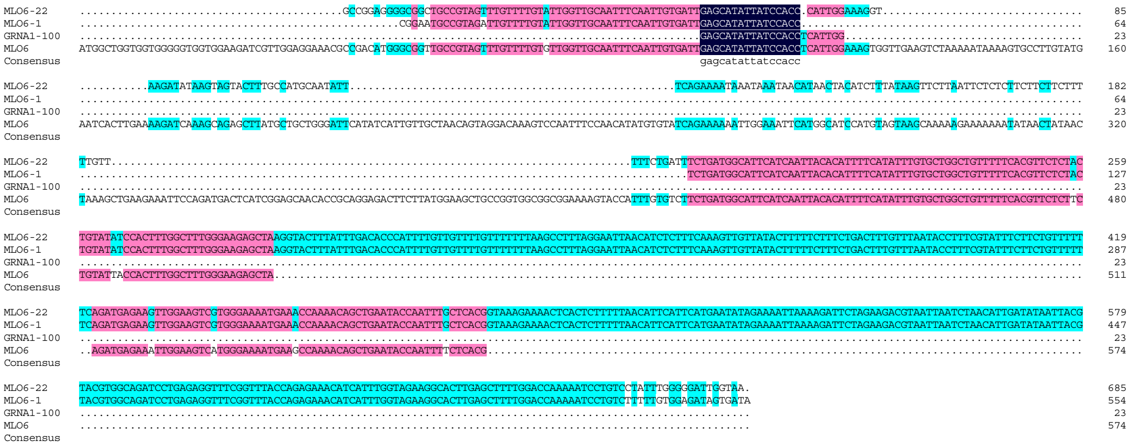

Supplementary Figure S16. Sequencing results of the CRISPR/Cas9-mediated *Lbmlo6* mutant lines 1 and 22.

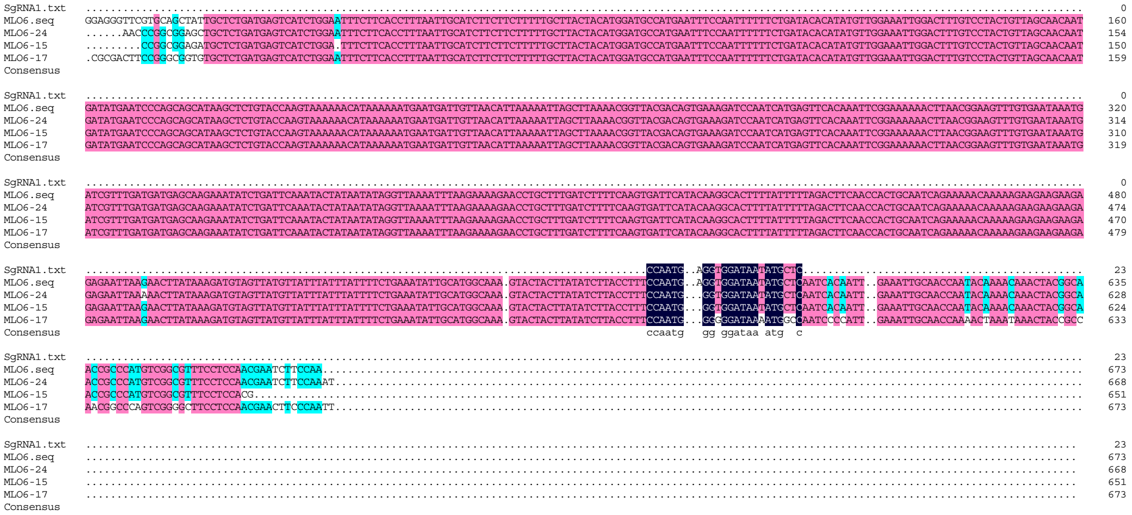

Supplementary Figure S17. Sequencing results of the CRISPR/Cas9-mediated *Lbmlo6* mutant lines 15, 17, and 24.

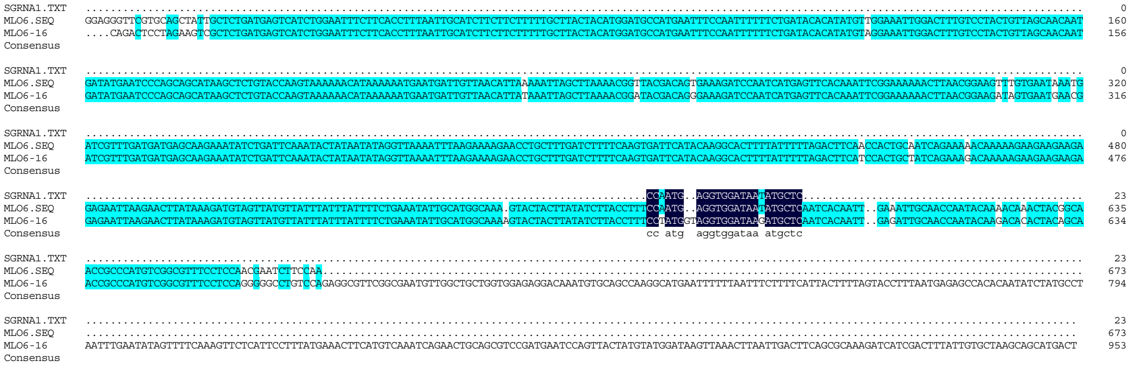

Supplementary Figure S18. Sequencing results of the CRISPR/Cas9-mediated *Lbmlo6* mutant line 16.
